# Recurrent Germline Variant in *RAD21* Predisposes Children to Lymphoblastic Leukemia or Lymphoma

**DOI:** 10.3390/ijms23095174

**Published:** 2022-05-05

**Authors:** Anne Schedel, Ulrike Anne Friedrich, Mina N. F. Morcos, Rabea Wagener, Juha Mehtonen, Titus Watrin, Claudia Saitta, Triantafyllia Brozou, Pia Michler, Carolin Walter, Asta Försti, Arka Baksi, Maria Menzel, Peter Horak, Nagarajan Paramasivam, Grazia Fazio, Robert J Autry, Stefan Fröhling, Meinolf Suttorp, Christoph Gertzen, Holger Gohlke, Sanil Bhatia, Karin Wadt, Kjeld Schmiegelow, Martin Dugas, Daniela Richter, Hanno Glimm, Merja Heinäniemi, Rolf Jessberger, Gianni Cazzaniga, Arndt Borkhardt, Julia Hauer, Franziska Auer

**Affiliations:** 1 Pediatric Hematology and Oncology, Department of Pediatrics, University Hospital Carl Gustav Carus, TU Dresden, 01307 Dresden, Germany; anne.schedel@uniklinikum-dresden.de (A.S.); ulrikeanne.friedrich@uniklinikum-dresden.de (U.A.F.); pia.michler@uniklinikum-dresden.de (P.M.); maria.menzel@uniklinikum-dresden.de (M.M.); meinolf.suttorp@uniklinikum-dresden.de (M.S.); 2 Department of Pediatrics, School of Medicine, Technical University of Munich; 80804 Munich, Germany; mina.morcos@tum.de (M.N.F.M.); f.auer@tum.de (F.A.); 3 Department of Pediatric Oncology, Hematology and Clinical Immunology, Heinrich-Heine University Duesseldorf, Medical Faculty, 40225 Duesseldorf, Germany; rabea.wagener@med.uni-duesseldorf.de (R.W.); titus.watrin@med.uni-duesseldorf.de (T.W.); triantafyllia.brozou@med.uni-duesseldorf.de (T.B.); sanil.bhatia@med.uni-duesseldorf.de (S.B.); arndt.borkhardt@med.uni-duesseldorf.de (A.B.); 4 Institute of Biomedicine, School of Medicine, University of Eastern Finland, Yliopistonranta 1, FI-70211 Kuopio, Finland; juha.mehtonen@uef.fi (J.M.); merja.heinaniemi@uef.fi (M.H.); 5 Tettamanti Research Center, Pediatrics, University of Milan Bicocca, Fondazione MBBM/San Gerardo Hospital, 20900 Monza, Italy; c.saitta2@campus.unimib.it (C.S.); g.fazio@hsgerardo.org (G.F.); gianni.cazzaniga@asst-monza.it (G.C.); 6 Institute of Medical Informatics, University of Muenster, 48149 Muenster, Germany; carolin.walter@uni-muenster.de (C.W.); martin.dugas@med.uni-heidelberg.de (M.D.); 7 Division of Pediatric Neurooncology, German Cancer Research Center (DKFZ), German Cancer Consortium (DKTK), 69120 Heidelberg, Germany; a.foersti@kitz-heidelberg.de (A.F.); robert.autry@kitz-heidelberg.de (R.J.A.); 8 Hopp Children’s Cancer Center Heidelberg (KiTZ), 69120 Heidelberg, Germany; 9 Institute of Physiological Chemistry, Medical Faculty Carl Gustav Carus, TU Dresden, 01307 Dresden, Germany; arka.baksi@tu-dresden.de (A.B.); rolf.jessberger@tu-dresden.de (R.J.); 10 Division of Translational Medical Oncology, National Center for Tumor Diseases (NCT) and German Cancer Research Center (DKFZ), 69120 Heidelberg, Germany; peter.horak@nct-heidelberg.de (P.H.); stefan.froehling@nct-heidelberg.de (S.F.); 11 Computational Oncology, Molecular Diagnostics Program, National Center for Tumor Diseases (NCT), 69120 Heidelberg, Germany; n.paramasivam@dkfz-heidelberg.de; 12 Institute for Pharmaceutical and Medicinal Chemistry, Heinrich-Heine-Universität Duesseldorf, Universitätsstraße 1, 40225 Duesseldorf, Germany; christoph.gertzen@hhu.de (C.G.); gohlke@uni-duesseldorf.de (H.G.); 13 John von Neumann Institute for Computing (NIC), Jülich Supercomputing Centre (JSC), Institute of Biological Information Processing (IBI-7: Structural Biochemistry), Forschungszentrum Jülich GmbH, 52425 Jülich, Germany; 14 Department of Clinical Genetics, University Hospital of Copenhagen, Faculty of health and Medical Sciences, University of Copenhagen, 2100 Copenhagen, Denmark; karin.wadt@regionh.dk; 15 Department of Paediatrics and Adolescent Medicine, Copenhagen University Hospital Rigshospitalet, 2100 Copenhagen, Denmark; kjeld.schmiegelow@regionh.dk; 16 Institute of Medical Informatics, Heidelberg University Hospital, 69120 Heidelberg, Germany; 17 Department of Translational Medical Oncology, National Center for Tumor Diseases (NCT) Dresden, 01307 Dresden, Germany; daniela.richter@nct-dresden.de (D.R.); hanno.glimm@uniklinikum-dresden.de (H.G.); 18 German Cancer Consortium (DKTK), 01307 Dresden, Germany; 19 Translational Functional Cancer Genomics, National Center for Tumor Diseases (NCT) and German Cancer Research Center (DKFZ), 69120 Heidelberg, Germany; 20 Medical Genetics, Department of Medicine and Surgery, University of Milan Bicocca, 20900 Monza, Italy; 21 German Cancer Consortium (DKTK), 81675 Munich, Germany

**Keywords:** acute lymphoblastic leukemia, trio sequencing, germline cancer predisposition, *RAD21*, cohesin complex

## Abstract

Somatic loss of function mutations in cohesin genes are frequently associated with various cancer types, while cohesin disruption in the germline causes cohesinopathies such as Cornelia-de-Lange syndrome (CdLS). Here, we present the discovery of a recurrent heterozygous *RAD21* germline aberration at amino acid position 298 (p.P298S/A) identified in three children with lymphoblastic leukemia or lymphoma in a total dataset of 482 pediatric cancer patients. While *RAD21* p.P298S/A did not disrupt the formation of the cohesin complex, it altered *RAD21* gene expression, DNA damage response and primary patient fibroblasts showed increased G2/M arrest after irradiation and Mitomycin-C treatment. Subsequent single-cell RNA-sequencing analysis of healthy human bone marrow confirmed the upregulation of distinct cohesin gene patterns during hematopoiesis, highlighting the importance of *RAD21* expression within proliferating B- and T-cells. Our clinical and functional data therefore suggest that *RAD21* germline variants can predispose to childhood lymphoblastic leukemia or lymphoma without displaying a CdLS phenotype.

## 1. Introduction

**Figure 1:**
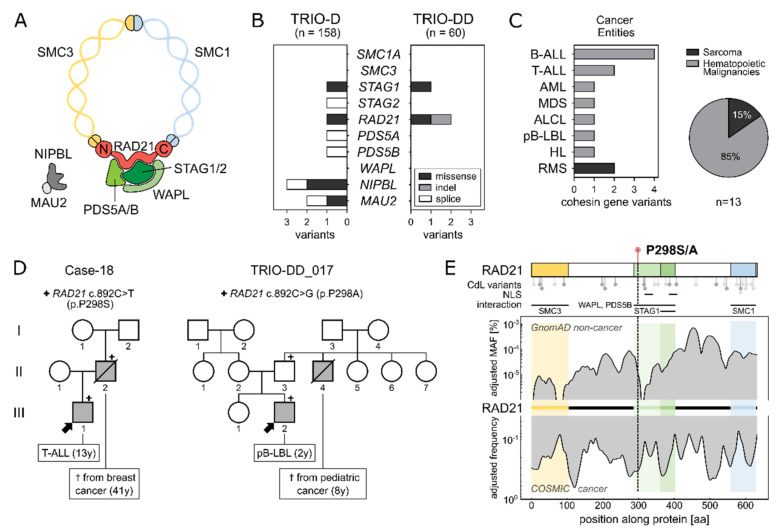
(**A**): The cohesin complex is formed by the 4 main core units SMC1 and SMC3 connected by RAD21 and STAG1 or STAG2. WAPL and PDS5 as co-factors and NIPBL and MAU2 as loaders are depicted. (**B**): Two patient cohorts (TRIO-D: *n* = 158 and TRIO-DD *n* = 60) were analyzed for germline variants within cohesin genes as depicted in Supplementary Table S1. Only non-synonymous variants with a MAF < 0.1% (gnomAD non-cancer population) were included. (**C**): Tumor entities of patients carrying a coding variant in one of the cohesin genes as shown in (**B**) (both cohorts combined, *n* = 13). Hematological malignancies account for 84.6% of cancers in the patients with germline cohesin variants. Further cohesin variants were identified in 2 patients with rhabdomyosarcoma. ALL: Acute lymphoblastic leukemia, AML: Acute myeloid leukemia, MDS: Myelodysplastic syndrome, ALCL: Anaplastic large-cell lymphoma, pB-LBL: precursor B-cell lymphoblastic lymphoma, HL: Hodgkin lymphoma, RMS: Rhabdomyosarcoma. (**D**): Family pedigrees of patients carrying the heterozygous germline *RAD21* variant p.P298S/A. Index patients are marked with an arrow. Family members affected by cancer are highlighted in grey. Variant carriers are marked with “+”. (**E**): Upper: RAD21 protein structure displaying the interaction domains with SMC3 (1-103 amino acids (AA)), WAPL and PDS5B (287-403AA), STAG1/STAG2 (362-403AA) and SMC1 (558-628AA, available online: http://genesdev.cshlp.org/content/23/18/2224.long accessed on 10 April 2022). Lollipops below depict the positions of variants known in Cornelia de Lange (CdL) syndrome patients, adapted from Krab et al. 2020, with light gray representing missense variants and in-frame deletions and darker gray representing protein truncations. Lower: Distribution of variant frequencies along RAD21, based on two databases: The top shows the adjusted MAF (%) of *RAD21* germline variants in the gnomAD non-cancer database, while the bottom shows the adjusted frequency of variants in the COSMIC (somatic cancer mutations) database.

The cohesin complex is one of the most essential keepers of genome stability, ensuring proper cell development and proliferation. Cohesin complex genes are ubiquitously expressed and are indispensable for cell survival [1]. Its central element is a highly conserved protein complex, formed as a ring-like structure by the helical proteins SMC1 and SMC3, which are in turn connected by RAD21 [2] and STAG 1/2 (also known as SA 1/2) [3,4] (Figure 1A). The co-factor WAPL is important for the cleavage in early phases of mitosis [5,6,7] and PDS5B can act both as maintenance and as a cohesin releasing factor [8]. Cohesin genes are first and foremost known for their involvement in chromatid aggregation and organized segregation in anaphase [9,10,11] with RAD21 cleavage marking the onset of anaphase [12]. Additionally, the complex participates in DNA double-strand break (DSB) repair, by holding the chromatids together during homologous recombination [13,14]. More recently, the cohesin complex has been implicated to govern the structure and function of chromatin. In this regard, the complex is involved in gene transcription through chromatid folding and RNA recruitment together with the CCCTC- binding factor (CTCF) [15,16], and has been shown to take part in the formation of topologically associated domains (TADs) [17].

*RAD21*-inactivating heterozygous somatic mutations are a well-established correlate of various human cancers, such as acute myeloid leukemia (AML) [18]. Furthermore, two cases with somatic truncating mutations in *RAD21* were recently identified in a study of pediatric precursor B-cell ALL (BCP-ALL) with very early relapse [19] and somatic cohesin mutations have been reported in pediatric high hyperdiploid leukemia [20]. Germline aberrations in cohesin complex genes are rare, but if present, cause syndromal disorders termed cohesinopathies. Cornelia-de-Lange syndrome (CdLS) is one of the best described examples, which exerts a condition of variable penetrance and expressivity presenting with neuro-developmental delays and abnormalities of the limbs [21]. While this syndrome is not typically known to confer cancer predisposition, an index case of a child with simultaneous occurrence of acute lymphoblastic leukemia (ALL) and CdLS caused by a *NIPBL* frameshift mutation has recently been reported [22]. Nevertheless, a possible link between additional germline cohesin complex gene mutations and childhood leukemia as well as cancer in general is still lacking. We find this quite surprising, given the established role of cohesins in various cancer types. Here, we describe a recurrent and functionally relevant mutated position within *RAD21* in three children with lymphatic malignancies originating from three different independent cancer cohorts.

## 2. Results

### 2.1. Identification of a Recurrent RAD21 Germline Alteration (p.P298S/A)

To add a novel piece to the understanding of cohesins in cancer predisposition, we analyzed whole exome sequencing data of an unselected German parent–child cohort of children with cancer (*n* = 60, TRIO-DD), as well as a recently published parent–child pediatric cancer cohort (*n* = 158, TRIO-D) [23] for germline variants in cohesin complex genes (Supplementary Table S1). Overall, in both childhood cancer cohorts, 13 variants (Minor allele frequency (MAF) < 0.1%; gnomAD non-cancer database) in seven different cohesin genes were identified (Figure 1B). All were transmitted from one of the parents, were mutually exclusive and significantly enriched in leukemia (lymphoid origin = 6, myeloid origin = 2) and lymphoma (*n* = 3) patients as compared to patients with solid tumors within the cohorts (Fisher’s exact test; *p* = 0.0081) (Figure 1C and Figure S1). Thereof, CdLS phenotypes were observed in one AML patient carrying *NIPBL* p.(G998E) (Case-92) and in one BCP-ALL patient harboring *MAU2* p.(N410S) (Case-74) (Supplementary Table S2). Nonetheless, none of the two patients presented with a definitive diagnosis of CdLS.

Interestingly, among all cohesin complex variants, one recurrently mutated nucleotide leading to an amino acid (AA) exchange at position 298 of *RAD21* (rs148308569) was identified in two families (one per cohort), in the absence of otherwise known-pathogenic variants (ClinVar) (Figures S2 and S3, Supplementary Tables S3 and S4). While the affected pediatric cancer patients carrying the recurrent *RAD21* variation did not show signs of CdLS, both three-generation pedigrees displayed a remarkable family history of early-in-life cancer (Figure 1D). In family I (Case-18), the heterozygous *RAD21* p.P298S (c.892C>T) variant was identified in a 13-year-old boy with T-ALL. His father, who transmitted *RAD21* p.P298S to his son, had died from breast cancer at the age of 41. Family II (TRIO-DD_017) displayed an alternative AA substitution at the same protein position (*RAD21* p.P298A; c.892C>G), which was detected in a 2-year-old patient with precursor B-cell lymphoblastic lymphoma (pB-LBL). Here, the variant was inherited from the healthy father, whose brother had died during childhood from cancer of unknown subtype (8y). 

*RAD21* p.P298 is evolutionarily conserved across species (GERP-score 5.61, phastCons = 1), located within the WAPL/PDS5B binding domain, and has not yet been reported in individuals with CdLS [24] (Figure 1E, Supplementary Table S5). While a low MAF at *RAD21* p.P298 and its surrounding AA indicates that these positions are rarely mutated in the germline of the non-cancer population (gnomAD database *n* = 118,479; MAF *RAD21* p.P298S < 10^−6^ and p.P298A < 10^−5^), high somatic variation frequencies (COSMIC database *n* = 37,221) are observed at the end of the SMC3 interaction domain and the start of the WAPL/PDS5B interacting domain, where the variants are located (Figure 1E). Furthermore, the CADD scores indicate potential deleterious effects with values of 22.3 and 22.5 for *RAD21* p.P298S and *RAD21* p.P298A, respectively. To assess the structural impact of *RAD21* p.P298S/A, we aimed to generate a computational model of the 50 adjacent residues on each side. However, several approaches failed to generate a secondary structure for this region, reflecting the substitution site as part of a very flexible and intrinsically disordered region (predicted disorder content of *RAD21*: 51.7%) (Figure S4).

### 2.2. RAD21 p.P298S/A Alters Cell Cycle and DNA Damage Responses

**Figure 2:**
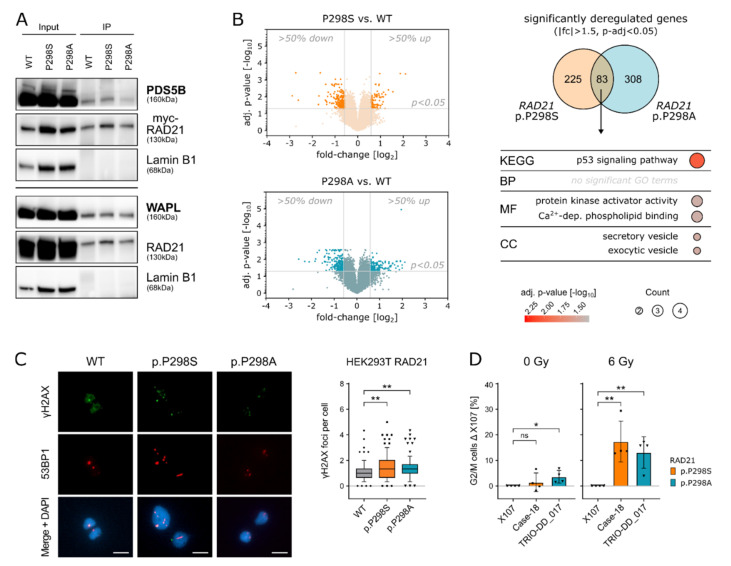
(**A**): Immunoprecipitation was performed on HEK293T cells overexpressing cMyc-tagged RAD21 WT, RAD21 p.P298S or RAD21 p.P298A. Cells were FCS-deprived and after 24 h arrested with colchicine (0.5 µg/mL) for 2 h, and the nuclear fraction was used for immunoprecipitation with the cMyc-tag. While the upper and lower panel represent one immunoprecipitation assay, they were run on two independent immunoblots and therefore presented as two panels. (**B**): Volcano plot of average gene expression based on microarray data. Fold-change and adjusted p-values are calculated by comparing *RAD21* p.P298S to WT (orange, top panel) and *RAD21* p.P298A to WT (blue, bottom panel). Probes with > 50% up- or downregulation and an adjusted *p*-value < 0.05 are considered as differentially expressed (DE) and highlighted in dark orange (*RAD21* p.P298S, top panel) or dark blue (*RAD21* p. P298A, bottom panel). DE genes are compared between *RAD21* p.P298S vs. WT and *RAD21* p.P298A vs. WT and show an overlap >20%. GO-term analysis of shared DE genes from the previous analysis identified enriched GO-terms. All GO-terms that exceed the significance (Benjamini–Hochberg FDR < 0.05) are represented. (**C**): Left: representative images of γH2AX (green) and 53BP1 (red) foci. DAPI (blue) was used for DNA labelling. Scale bar: 10 µm. Right: quantification of γH2AX foci per cell in HEK293T *RAD21* WT, p.P298A and p.P298S cells. Experiments were performed as 3 independent replicates. Values are expressed in boxplots with whiskers from percentile 10–90. For the statistical analysis, Student’s *t*-test was performed (** = *p* ≤ 0.01). (**D**): X107 (healthy control, *RAD21* WT), Case-18 (*RAD21* p.P298S), and TRIO-DD_017 (*RAD21* p.P298A) primary fibroblasts were subjected to irradiation with 6 Gy (*n* = 4) and the cell cycle analyzed using propidium iodide staining. For indicated *p*-values, Student’s *t*-testing was performed (* = *p* ≤ 0.05; ** = *p* ≤ 0.01). Case-18 and TRIO-DD_017 were adjusted to X107 as a baseline response.

Given that *RAD21* p.P298S/A is located in a hyper-flexible domain, we next aimed to investigate its interaction with cohesin complex partners. Therefore, the identified *RAD21* variants were cloned and transfected into HEK293T cells (R32-hRAD21). In analogy to *RAD21* WT, neither protein expression nor nuclear localization were affected by the variants *RAD21* p.P298S/A (Figure S5). Immunoprecipitation assays of the nuclear fraction showed binding of RAD21 with WAPL and PDS5B for the WT, as well as for both mutant proteins RAD21 p.298S/A, respectively (Figure 2A). Furthermore, the interaction of RAD21 WT and RAD21 p.P298S/A to SMC1 and STAG2 were comparable (Figure S6), suggesting that RAD21 p.P298S/A does not perturb the formation of the cohesin complex. 

Since one additional function of the complex is the control of transcriptional regulation through genome-wide chromatin organization [25,26], we next tested the effect of *RAD21* p.P298S/A on gene expression by microarray analysis in the cell line system described above. Hierarchical clustering of differentially expressed genes (|fc| >1.5, adj. *p*-value < 0.05) showed a clear clustering of replicates and a separation of each condition (Figure S7). In total, 308 and 391 genes were differentially regulated (|fc| > 1.5, adj. *p*-value < 0.05) in cells carrying the *RAD21* variants p.P298S/A, respectively. A total of 83 genes were significantly up-/down-regulated in both *RAD21* cell line models (Figure 2B and Figure S8, Supplementary Table S6). GO term analysis of these genes identified “p53 signaling pathway” as the most prominent among enriched deregulated signaling pathways (Figure 2B). In line with these observations, HEK293T cells carrying *RAD21* p.P298S/A showed an increased number of γH2AX and 53BP1 co-localized foci indicating the extent of DNA double-strand breaks resulting from the mutated RAD21 protein compared to the WT (** = *p* ≤ 0.01; Student’s *t*-test) (Figure 2C).

Based on these results, we questioned whether patients carrying *RAD21* p.P298S/A would also display DNA damage signaling abnormalities during normal and cellular stress conditions. Therefore, primary patient fibroblasts carrying the respective *RAD21* p.P298S/A variants in comparison to *RAD21* WT control fibroblasts were challenged through irradiation to induce DNA damage and their response assessed via cell-cycle analysis. Both fibroblastic cell lines carrying *RAD21* p.P298A and *RAD21* p.P298S displayed a significant G2/M cell-cycle arrest compared to a WT control after ionizing irradiation (Figure 2D and Figure S9). Likewise, upon treatment with the DNA cross-linking agent Mitomycin-C (MMC), *RAD21* p.P298S fibroblasts arrested more cells at the S/G2/M cell-cycle stage (*p* = 0.0033; Student’s *t*-test) (Figure S10). Therefore, the observed G2/M cell cycle arrest is a potential phenotype of the increased DNA damage occurring in cells carrying *RAD21* p.P298S/A upon exposure to stress conditions and further underlines the increased risk of malignant transformation for predisposed patients.

### 2.3. Amino Acid Replacements (S/A) at Position 298 of RAD21 Lead to Altered RAD21 Expression Levels

**Figure 3:**
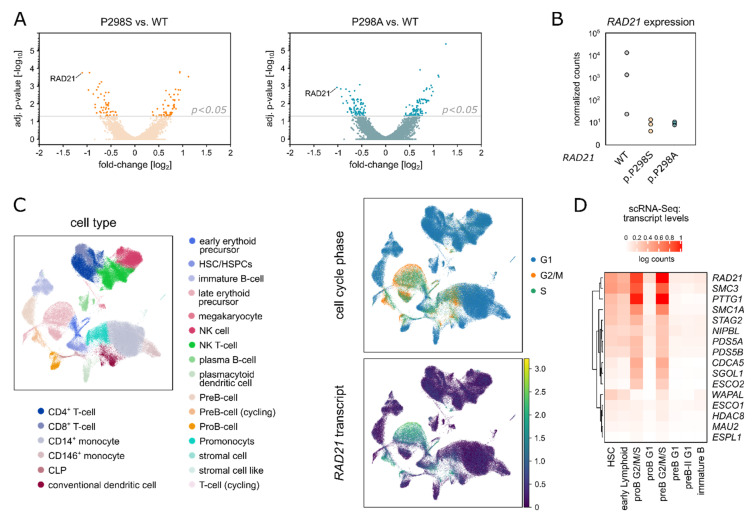
(**A**)**:** Volcano plot of average gene expression based on bulk RNA-Sequencing data. Fold-change and adjusted *p*-values are calculated by comparing *RAD21* p.P298S to WT (orange, left panel) and *RAD21* p.P298A to WT (blue, right panel). Genes with an adjusted *p*-value < 0.05 are considered as differentially expressed and highlighted in dark orange (*RAD21* p.P298S, left panel) or dark blue (*RAD21* p.P298A, right panel). (**B**): Expression of *RAD21*, as the top down-regulated gene in both *RAD21* variants, is separately indicated for *RAD21* WT, p.P298S and p.P298A (three biological replicates each, bulk RNA-Sequencing). (**C**): Left: UMAP-visualization of the healthy human bone marrow scRNA-seq data. Right: Cell cycle stages colored on the UMAP-visualization (upper) and *RAD21* gene expression colored on the UMAP-visualization (lower). (**D**): Heat map indicating the cohesin complex genes’ expression levels in cells of the different stages of B-cell differentiation.

To elucidate the molecular mechanism of *RAD21* dysregulation mediated through both variants, we employed an additional variant specific model by generating a HEK293T cell line with doxycycline-inducible expression of siRNA targeting the endogenous *RAD21* and concomitant expression of EGFP-tagged pRTS-1-*RAD21* WT, p.P298A or p.P298S [27]. Three days after doxycycline induction, cells of each condition were EGFP-sorted and subjected to RNA-Sequencing (Figure S11A). In parallel, endogenous RAD21 downregulation and its replacement by EGFP-tagged RAD21 was verified by Western Blot analysis (Figure S11B), while the presence of the respective *RAD21* variants was additionally validated by Sanger Sequencing (Figure S11C). In total, the RNA-Sequencing yielded only 50 commonly deregulated genes between both variants and RAD21 WT (Figure S12, Supplementary Table S7) (adj. *p*-value < 0.05). These results are in line with published data confirming only modest gene expression changes with mostly weak effects observed immediately upon cohesin loss [28]. Nevertheless, *RAD21* itself ranked as the top downregulated gene for both, the *RAD21* p.P298A and the *RAD21* p.P298S variant conditions, compared to the WT *RAD21* cells (Figure 3A,B). Therefore, these data provide evidence that the here identified amino acid replacements at position 298 of RAD21 confer a functional effect in hampering proper *RAD21* transcription levels. 

Thus, to identify vulnerable populations during hematopoietic differentiation, which are dependent on high *RAD21* expression and would be potentially susceptible to *RAD21* p.P298S/A, single-cell RNA-Sequencing (scRNA-Seq) data of healthy human bone marrow from the Human Cell Atlas were analyzed for cohesin complex gene expression. In line with its essential role in mitosis, *RAD21* expression was primarily up-regulated in actively dividing cells within the G2/M or S-phase compared to cells in G1 (*p* < 2.2 × 10^−16^, Wilcoxon test) (Figure 3C and Figure S13). Particularly high *RAD21* transcript levels clustered with *SMC3* and *PTTG1* transcripts and were detected in cycling pre- and pro-B-cells, while *RAD21* expression in common lymphoid progenitors (CLPs) and hematopoietic stem and progenitor cells (HS/PCs) was significantly lower (*p* < 2.2 × 10^−16^, Wilcoxon test) (Figure 3D and Figure S14). These data are in line with the expression pattern of *RAD21* in human leukemias, as observed in gene and protein expression data across various hematological malignancies (Figure S15). 

### 2.4. RAD21 p.P298S/A Is Recurrently Found in Pediatric Lymphoblastic Leukemia/Lymphoma

To confirm a correlation between germline *RAD21* p.P298S/A and pediatric leukemia, we analyzed an additional unpublished pediatric cancer cohort of 150 children with relapsed ALL (Italian IntReALL standard risk study; R-ALL) for *RAD21* p.P298S/A. Here, we identified a third case with *RAD21* p.P298A in a boy who was diagnosed with B-cell precursor ALL (BCP-ALL) at 12 years old and had a combined bone marrow/CNS relapse 5 years later (Table 1). In a fourth cohort including 114 children and adolescents with therapy refractory leukemia and lymphoma (INFORM), no germline indels or missense variants affecting *RAD21* were identified, suggesting no enrichment in the relapsed or therapy refractory patients. To further cross-validate *RAD21* p.P298S/A in a non-pediatric cancer setting, a cohort of 2300 young adults (<51 years) with cancer was mined (MASTER program). In this extensive sample collection, only one patient harboring *RAD21* p.P298A with a solid tumor was identified (Table 1). Therefore, amongst all cohorts, *RAD21* p.P298S/A was found to be enriched in pediatric vs. adult cancers (3/482 vs. 1/2300; Fisher’s exact test; *p* = 0.018). Overall, we did not observe an enrichment in the relapsed or therapy refractory patient cohorts suggesting that *RAD21* p.P298S/A predisposes to lymphoid precursor malignancies with no influence on therapy response.

## 3. Discussion

The cohesin complex is a cogwheel of ordered chromosome alignment and segregation during cell division, homologous-recombination-driven DNA repair and regulation of gene expression [5,29,30]. RAD21 is essential for this machinery as it connects the SMC1 and SMC3 cohesin subunits and thereby generates the functional ring-like structure of cohesin

Overall, within all analyzed datasets, comprising in total 482 pediatric cancer patients and 2300 adult cancers as controls, we present three children with lymphoblastic leukemia/lymphoma all carrying a recurrent *RAD21* germline variation at position 298. None of the patients displayed a CdLS phenotype, which is in line with previous reports, showing that *RAD21* variants are known to display reduced CdLS phenotype expressivity [24]. Furthermore, as with other *RAD21* missense variants in cancer [31], the here identified *RAD21* p.P298S/A alterations are heterozygous and mutually exclusive to other variants in cohesin complex genes. 

The observed familial cancer history in two of the patients demonstrates an increased cancer risk across generations. Nevertheless, due to the incomplete penetrance and the tumor variance, additional factors such as synergizing germline mutations or environmental influences to drive tumor evolution need to be taken into account. Interestingly, in two patients carrying *RAD21* p.P298S/A we identified a known pathogenic KRAS hot-spot mutation as a common somatic denominator in the respective tumors, which is in line with a recently published association between cohesin complex mutations and RAS signaling in cancer progression [32]. 

Functionally, the described alterations at position 298 did not disturb the formation of the cohesin complex, which is also rarely seen in variants without detrimental gene disruption [33]. Mechanistically, we could show that the described variants caused deregulations of proper *RAD21* transcript levels, which in the long-term affected p53 signaling. By applying irradiation and MMC as external stressors this effect was further enhanced as seen by increased cell cycle arrest in primary patient cells carrying *RAD21* p.P298S/A. Likewise, *RAD21* variants have been previously described in radiosensitive cancer patients [34] and CdLS patients displaying increased DNA damage sensitivity [35,36]. Furthermore, embryonic stem cells of *RAD21* heterozygous mice show significantly reduced survival after treatment with MMC [30]. Thus, the increased G2/M arrest in germline cells carrying *RAD21* p.P298S/A emphasizes the crucial role of properly functioning cohesins to avoid chromosomal instabilities during the repair of both interstrand MMC-DNA cross-links [37] and irradiation-induced DNA DSB [14,38].

Although cohesin complex genes are supposed to be ubiquitously expressed owing to their inevitability for basic cellular processes, we utilized scRNA-Seq to newly demonstrate that cohesin complex partners are differentially regulated during B-cell lineage specification in human bone marrow. Even though HS/PCs require cohesin, *Rad21* haploinsufficiency in mice was postulated to display distinct hematopoietic phenotypes in comparison to other cohesin subunit knockout models [39], further supporting the here described cohesin gene specific expression patterns during early B-cell differentiation. Interestingly, high expression of WAPL was identified particularly in HS/PCs, pointing towards a so far unrecognized role of WAPL within the stem cell compartment. *STAG2*, *RAD21*, *SMC3* and *SMC1* loss of function is known to induce stemness potential such as enhanced self-renewal and differentiation arrest in human and mouse HS/PCs [33,40]. Along these lines, it was also shown that cohesin facilitates V(D)J recombination in pro-B cells [41] and T-cell receptor α locus rearrangement [42]. 

Moreover, cohesins and their associated proteins are being recognized to act as master transcriptional regulators of hematopoietic genes [43]. Therefore, their deregulation can be regarded as a critical first step in the evolution of hematopoietic malignancies [40,44]. Intriguingly, the here identified patients harboring *RAD21* p.P298S/A all suffered from precursor lymphoblastic malignancies, which suggests either stem and progenitor cells or early lymphoid precursors as the origins of the disease. 

Taken together, in addition to *RAD21* germline and somatic loss-of-function variants that result in cohesinopathies and predominantly myeloid cancers, respectively, our data propose a third category of *RAD21* variants that mediate germline predisposition to lymphoblastic malignancies in childhood. Understanding the influence of *RAD21* germline variants may offer new treatment options such as their potential sensitivity to PARPP inhibitors which are already included in clinical trials in leukemias with somatically mutated cohesin [45].

## 4. Materials and Methods

### 4.1. Patients

Patients ≤ 19 years of age were unselectively recruited at the Pediatric Oncology Department, Dresden (years 2019–2020), or as previously described [23,46,47]. Consent of the families was obtained according to the Ethical Vote EK 181042019 (Dresden) and in line with the Declaration of Helsinki. For the IntReALL cohort, patients’ parents or their legal guardians gave informed consent to genetic analyses in the context of add-on studies linked to the clinical protocol to which patients were enrolled. 

### 4.2. Whole Exome Sequencing (WES)

Germline DNA was extracted from the patient’s fibroblasts using AllPrep DNA/RNA Mini Kit (Qiagen, Venlo, Netherlands) and from PBMCs of the parents and the remaining patient’s using the QIAamp DNA Blood Mini Kit (Qiagen). Sequenceable next-generation libraries for WES were generated with the SureSelect Human All Exon V7 kit (Agilent Technologies, Santa Clara, California, USA). The libraries were sequenced on a NovaSeq 6000 platform (Illumina, San Diego, CA, USA) in paired-end mode (2 × 150bp) and with final on-target coverage of ≥100×. Processing of the WES data was performed as previously described [23].

### 4.3. Cell Culture

Primary fibroblasts were initially cultivated in BIO-AMF™-2 Medium (Biological-Industries, Kibbutz Beit Haemek, Israel) up to a passage of 5. For experimental analysis, fibroblasts were cultured in Dulbecco’s Modified Eagle Medium (DMEM; GIBCO/Thermo Fisher Scientific, Waltham, Massachusetts, USA) with 20% fetal calf serum (FCS; GIBCO), 1% Penicillin/Streptomycin (P/S; 10,000 units/mL; GIBCO) and 1% MEM Non-essential Amino Acids (NEAA; GIBCO) up to a passage of 13.

HEK293T cells transfected with R32-hRAD21 were cultured in DMEM with 10% FCS, 1% P/S and 1% NEAA. All cells were kept at 37 °C and 5% CO_2_. 

### 4.4. Cloning

The inducible RAD21 system (pRTS-1-RAD21) was gifted from Kerstin Wendt and Olaf Stemman [27]. Mutated cDNAs for *RAD21* p.P298A and p.P298S were created by site directed mutagenesis by PCR and cloned into the pMC3.Hygro (=R32-hRAD21) and the pRTS-1 (=pRTS-1-RAD21) plasmid via MluI/SpeI and SwaI/XhoI restriction sites, respectively, utilizing the following primer pairs (Table 2):

### 4.5. HEK293T Cell Transfection

R32-hRAD21

HEK293T cells were seeded at a density of 4x10^5^ cells and stably transfected with 4 µg of Vector [48] (R32-hRAD21 or R32-hRAD21 p.P298S or R32-hRAD21 p.P298A using Lipofectamine2000 (Invitrogen) and selected with Hygromycin (Invitrogen/Thermo Fisher Scientific, Waltham, MA, USA) at a concentration of 200 µg/mL for 7 days. Continuous culturing was performed with Hygromycin concentration altering between 100 µg/mL and 200 µg/mL, put freshly 3 times a week.

pRTS-1-RAD21

HEK293T cells were seeded at a density of 5x10^5^ cells and stably transfected with 4 µg of Vector [27] (pRTS-1-RAD21, pRTS-1-RAD21 p.P298S or pRTS-1-RAD21 p.P298A using Lipofectamine2000 (Invitrogen) and selected with Hygromycin (Invitrogen) at a concentration of 400 µg/mL for 7 days. Continuous culturing was performed with Hygromycin at concentrations altering between 200 µg/mL and 400 µg/mL, put freshly 3 times a week.

### 4.6. Microarray (R32-hRAD21)

Stably transfected HEK293T cells overexpressing R32-hRAD21 with either WT, p.P298S or p.P298A conditions were seeded onto 10 cm plates in a density of 2 × 10^6^ cells in quadruplicates. After 48 h, control cells were harvested and 6 × 10^6^ cells were pelleted and stored at −80 °C for later RNA extraction. RNA was extracted using the RNeasy Mini Kit (Qiagen #74106) with 350 µL of RLT Buffer+ BME using QIAshredder (#79656) and RNAse-Free DNase Set (Qiagen #79254). RNA was stored at −80 °C.

RNA samples were sent to Macrogen Europe B.V. (Amsterdam, Netherlands) for gene expression analysis using the SurePrint G3 Human Gene Expression 8 × 60K v3 microarray (Agilent, Inc., Santa Clara, CA, USA). Put briefly, Cy3-labeled cRNA was prepared from 1~5 µg total RNA (Quick Amp Labeling Kit, Agilent), subsequently fragmented and (1.65 µg) hybridized to the microarray. Scanning was performed by the SureScan Microarray Scanner System G4900DA (Agilent).

For analysis, raw data were extracted using the software provided by Agilent Feature Extraction Software (v11.0.1.1). The raw data for the same probe was summarized automatically in the Agilent feature extraction protocol to provide expression data for each gene probed on the array. Flag A-tagged probes were filtered out and the remaining gProcessedSignal values were log transformed and quantile normalized. 

Furthermore, all technical replicates (*n* = 4) of one sample were combined and samples were compared pairwise by fold-change values: *RAD21* p.P298A vs. WT, *RAD21* p.P298S vs. WT and *RAD21* p.P298A vs. *RAD21* p.P298S. The *p*-value calculated with an independent Student’s *t*-test was corrected for multiple testing and used to define the significance of these pairwise comparisons. Genes with an absolute fold-change of 1.5 or more and an adjusted *p*-value below 0.05 were considered as significantly up- or down-regulated. These data (*n* = 995 probes) were used to perform a two-dimensional hierarchical clustering using Euclidean distance and complete linkage. Results were represented as heat map (seaborn.clustermap v.0.10.1 with prior optimal leaf ordering, Python v.3.6). The same analysis was performed for a smaller set (*n* = 83 probes), which were differentially expressed in both mutants *RAD21* p.P298A and p.P298S vs. WT was similarly analyzed and represented.

### 4.7. Quantitative Real-Time (qRT)-PCR Analysis

RNA was extracted from primary fibroblasts (TRIO_DD_018; TRIO_DD_025; 2.0–3.0 × 10^6^ cells) using the RNaeasy Mini Kit (Qiagen #74106) with 350 µL of RLT Buffer+ beta-ME using QIAshredder (#79656) and RNAse-Free DNase Set (Qiagen #79254). A total of 3 independent RNA extractions were performed, and 1 µg of RNA was reverse transcribed into cDNA using the QuantiTect Reverse Transcription Kit (Quiagen #205311) following manufacturer’s instructions. The qRT-PCR was performed using TaqMan Universal Master Mix II following manufacturer’s instructions (Thermo Fisher Scientific, Waltham, MA, USA, #PN4428173) for 20 µL reaction with 1.5 µL of cDNA. The following TaqMan assays were used: TBP (Hs00427620_m1), HPRT1 (Hs02800695) and POT1 (Hs00209984_m1). Expression of mRNA was analysed by the comparative ΔΔ-C_T_ method and plotted in relation to the control sample.

### 4.8. GO-Term Analysis

Gene Ontology (GO) term analysis was performed using the web server EnrichR (https://maayanlab.cloud/Enrichr/; accessed on 13 April 2021) [49]. GO terms of the categories “Molecular Function”, “Biological Pathway”, “Cellular Component” and “KEGG” were analyzed and results with an adjusted *p*-value < 0.05 are represented.

### 4.9. Cell Sorting and RNA-Sequencing (pRTS-1-RAD21)

HEK293T pRTS-1-RAD21 cells stably selected with Hygromycin, were induced with Doxycycline at a concentration of 2 µg/mL for 72 h. All cells were trypsinized, and washed with cold PBS. Cells were diluted in cold FACS Buffer (PBS + 2 µM EDTA) and kept on ice until sorting. Cell sort for high EGFP was performed on an FACSAria II (BD). 

RNA Extraction was performed using the RNA Micro Kit (Qiagen) following manufacturer’s instruction. RNA quality analysis was performed on an Agilent 2100 bioanalyzer, with all samples showing RIN values of 10. RNA libraries were prepared by mRNA enrichment by poly-dT pull down using the NEBNext Poly(A) kit based on manufacturer’s recommendations (New England Biologies, Ipswitch, MA, USA). Sequencing was carried out as 2 × 50 bp reads and read depths of 30–50 million on an Illumina NovaSeq 6000.

FastQC (v.0.11.9; http://www.bioinformatics.babraham.ac.uk/, accessed on 10 April 2022) was used to perform a basic quality control of the resulting sequencing data. Fragments were aligned to the human reference genome hg38 with support of the Ensembl 104 splice sites using the aligner gsnap (v2020-12-16) [50]. Counts per gene and sample were obtained based on the overlap of the uniquely mapped fragments with the same Ensembl annotation using featureCounts (v2.0.1) [51]. The normalization of raw fragments based on library size and testing for differential expression between the different cell types/treatments was performed with the DESeq R package (v1.30.1) [52]. Sample to sample Euclidean distance, Pearson and Spearman correlation coefficients (r) and PCA based upon the top 500 genes showing highest variance were computed to explore correlation between biological replicates and different libraries. To identify differentially expressed genes, counts were fitted to the negative binomial distribution and genes were tested between conditions using the Wald test of DESeq2. Resulting p-values were corrected for multiple testing with the Independent Hypothesis Weighting package (IHW 1.12.0) [53]. Genes with a maximum of 5% false discovery rate (padj ≤ 0.05) were considered as significantly differentially expressed.

### 4.10. Statistical Analyses

For statistical analysis, the two-tailed Student’s unpaired t-test was performed. Differences with a *p* value < 0.05 were considered to be significant, ns = *p* > 0.05, * = *p* ≤ 0.05, ** = *p* ≤ 0.01, *** = *p* ≤ 0.001, **** = *p* ≤ 0.0001.

## Figures and Tables

**Table 1:** Cohort descriptions and identified RAD21 variants analyzed in context of clinical phenotypic and pathogenic findings. HR = High risk, SR = Standard risk, N/A = not applicable, pB-LBL = B-cell lymphoblastic lymphoma, T-ALL = T-cell acute lymphoblastic leukemia, BCP-ALL = precursor B-cell acute lymphoblastic leukemia, MPNST = Malignant peripheral nerve sheath tumor.

		TRIO-DD	TRIO-D	R-ALL	INFORM	MASTER
Cohort	Number of patients	***n* = 60**	***n* = 158**	***n* = 150**	***n* = 114**	***n* = 2300**
		pediatric	pediatric	pediatric	pediatric	adult
	% Hematopoietic malignancies	38.3%	51.3%	100%	100%	3.7%
	Inclusion criteria	Primary diagnosis	Primary diagnosis	IntReALL SR	Therapy refractory	Young adults < 51 y
Patient	Sex	Male	Male	Male	-	Female
	Age	2	13	12	-	53
	Tumor	pB-LBL	T-ALL	BCP-ALL	-	MPNST
	Risk group	SR	HR	SR	-	N/A
*RAD21* variant p.P298	Protein exchange	ENSP00000297338.2 p.P298A	ENSP00000297338.2 p.P298S	ENSP00000297338.2 p.P298A	-	ENSP00000297338.2 p.P298A
	Base exchange	ENST00000297338.2 c.892 C>G	ENST00000297338.2 c.892 C>T	ENST00000297338.2 c.892 C>G	-	ENST00000297338.2 c.892 C>G
	SNP ID	rs148308569	rs148308569	rs148308569	-	rs148308569
	MAF GnomAD	10^−5^	10^−6^	10^−5^	-	10^−5^
	MAF within the cohort	1.7 × 10^−2^	6.5 × 10^−3^	6.7 × 10^−3^	-	4.3 × 10^−4^
Genetic history	Genetic counselling *	+	+	unknown	-	unknown
	Family history	+	+	unknown	-	unknown
2nd Hit	Somatic Mutations	unknown	*KRAS* p.Q61R	*KRAS* p.G12C	-	*PTCH2* p.A68V

SR = standard risk, HR = high risk, * based on criteria from Jongmans et al. Eur J Med Genet 59 (2016) 116-125 und Ripperger et al., Am J Med Genet A. (2017).

**Table 2:** Primer sequences for cloning.

Name	Sequence (5′ → 3′)
hRad21_MluI_F	GGCGCacgcgtgccaccATGTTCTACGCACATTTTGTTCTC
hRad21_SpeI_R	CCTCGactagtTATAATATGGAACCTTGGTCCAGGTGTTGC
hRad21_SwaI_F	GGCGCATTTAAATCATGTTCTACGCAC
hRad21_XhoI_R	CCTCGCTCGAGTCCATATAATATGGAACC
hRad21_P298S_F	GATCAAACAACACTTGTTtCAAATGAGGAAGAAGCATTTGC
hRad21_P298S_R	GCAAATGCTTCTTCCTCATTTGaAACAAGTGTTGTTTGATC
hRad21_P298A_F	GATCAAACAACACTTGTTgCAAATGAGGAAGAAGCATTTGC
hRad21_P298A_R	GCAAATGCTTCTTCCTCATTTGcAACAAGTGTTGTTTGATC

## Data Availability

The *RAD21* variant was submitted to ClinVar (https://www.ncbi.nlm.nih.gov/clinvar/, accessed on 4 May 2022). The datasets generated during and/or analysed during the current study are available from the corresponding author on reasonable request.

## Supplementary Materials

The following are available online at https://www.mdpi.com/article/10.3390/ijms23095174/s1.
