# Supplementary material for: Recurrent Germline Variant in RAD21 Predisposes Children to Lymphoblastic Leukemia or Lymphoma
Source: Int J Mol Sci. 2022 May 5;23(9):5174. doi: 10.3390/ijms23095174 (PMC9106003; doi:10.3390/ijms23095174)
Supplement: Supplementary file 1 [file ijms-23-05174-s001.zip › ijms-1701368-supplementary/Schedel et al_Supplementary Figures.pptx]

## Slide 1
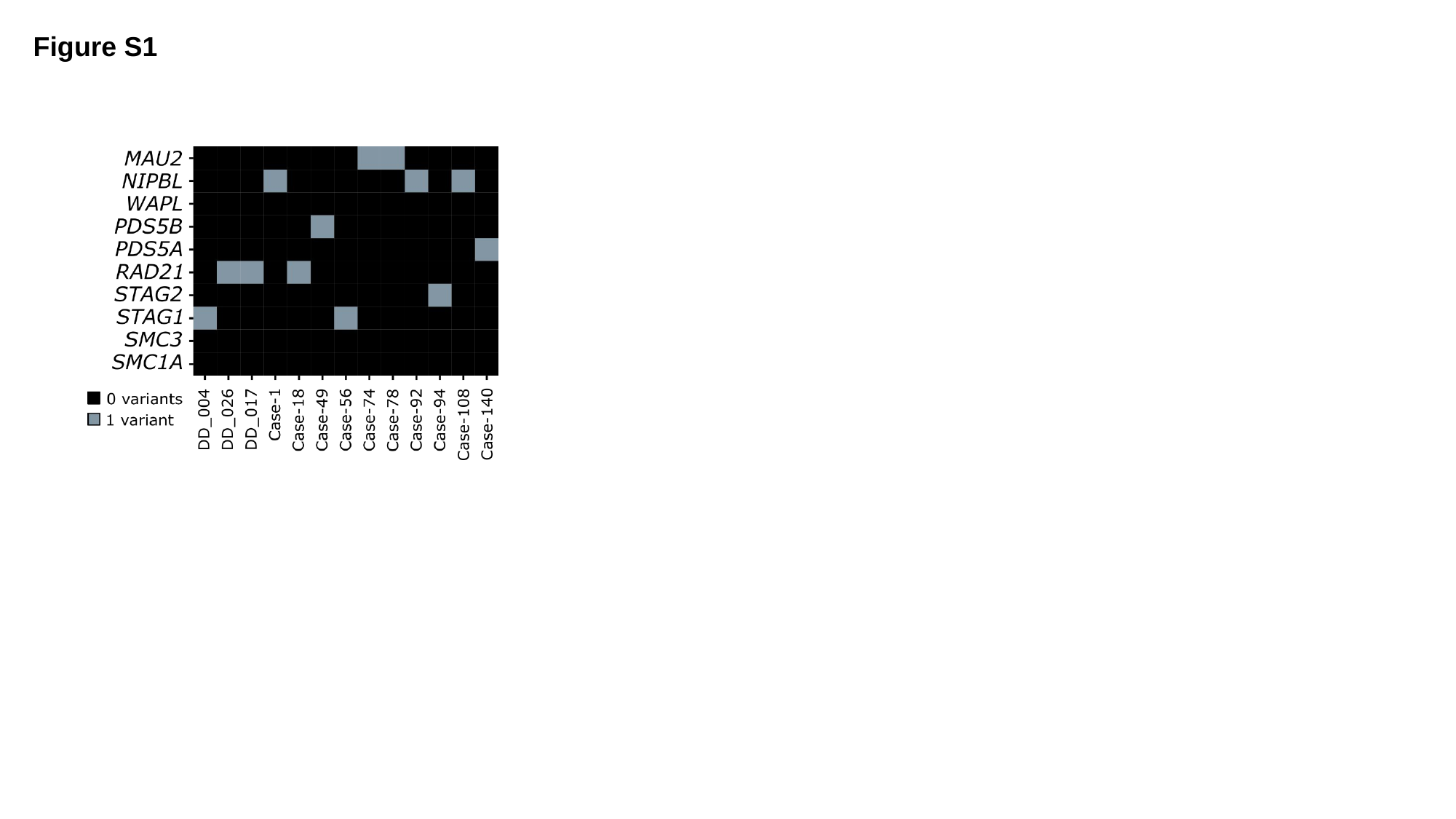

Figure S1

## Slide 2
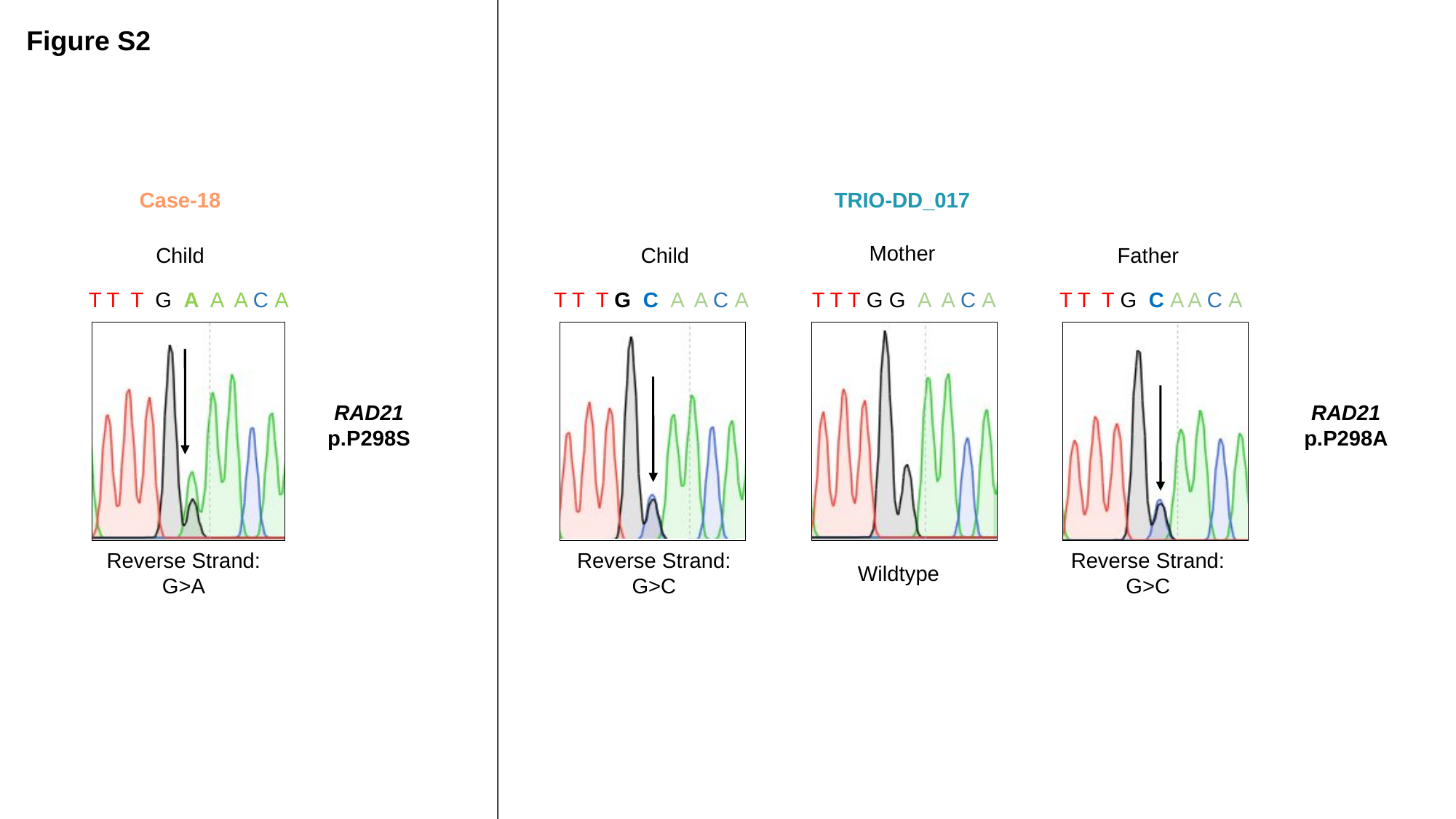

Figure S2
TRIO-DD_017
Mother
Child
Father
T T T G C A A C A
RAD21 p.P298A
Reverse Strand: G>C
Wildtype
Reverse Strand: G>C
Case-18
Child
T T T G A A A C A
T T T G G A A C A
T T T G C A A C A
RAD21 p.P298S
Reverse Strand: G>A

## Slide 3
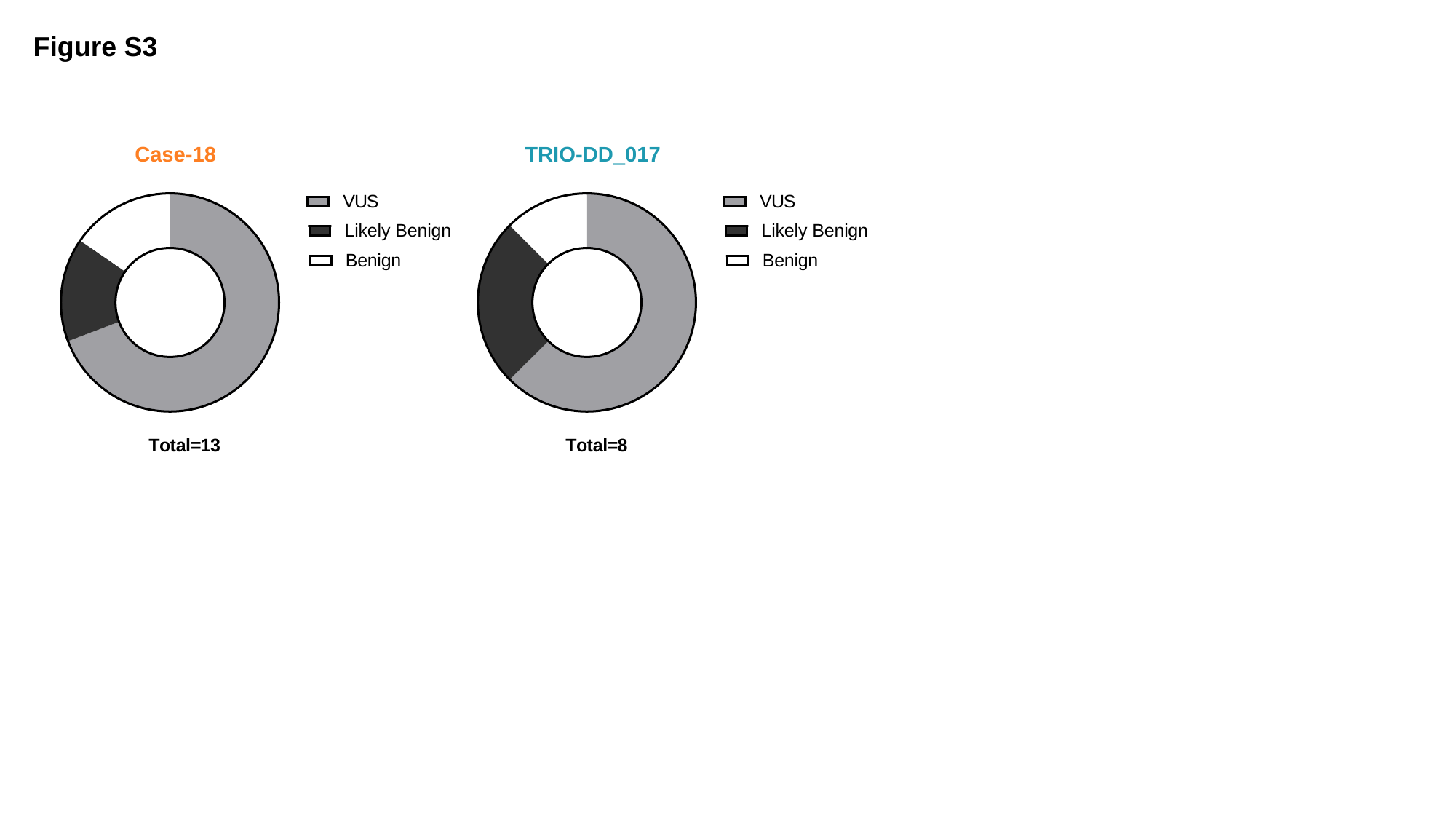

Figure S3
Case-18
TRIO-DD_017

## Slide 4
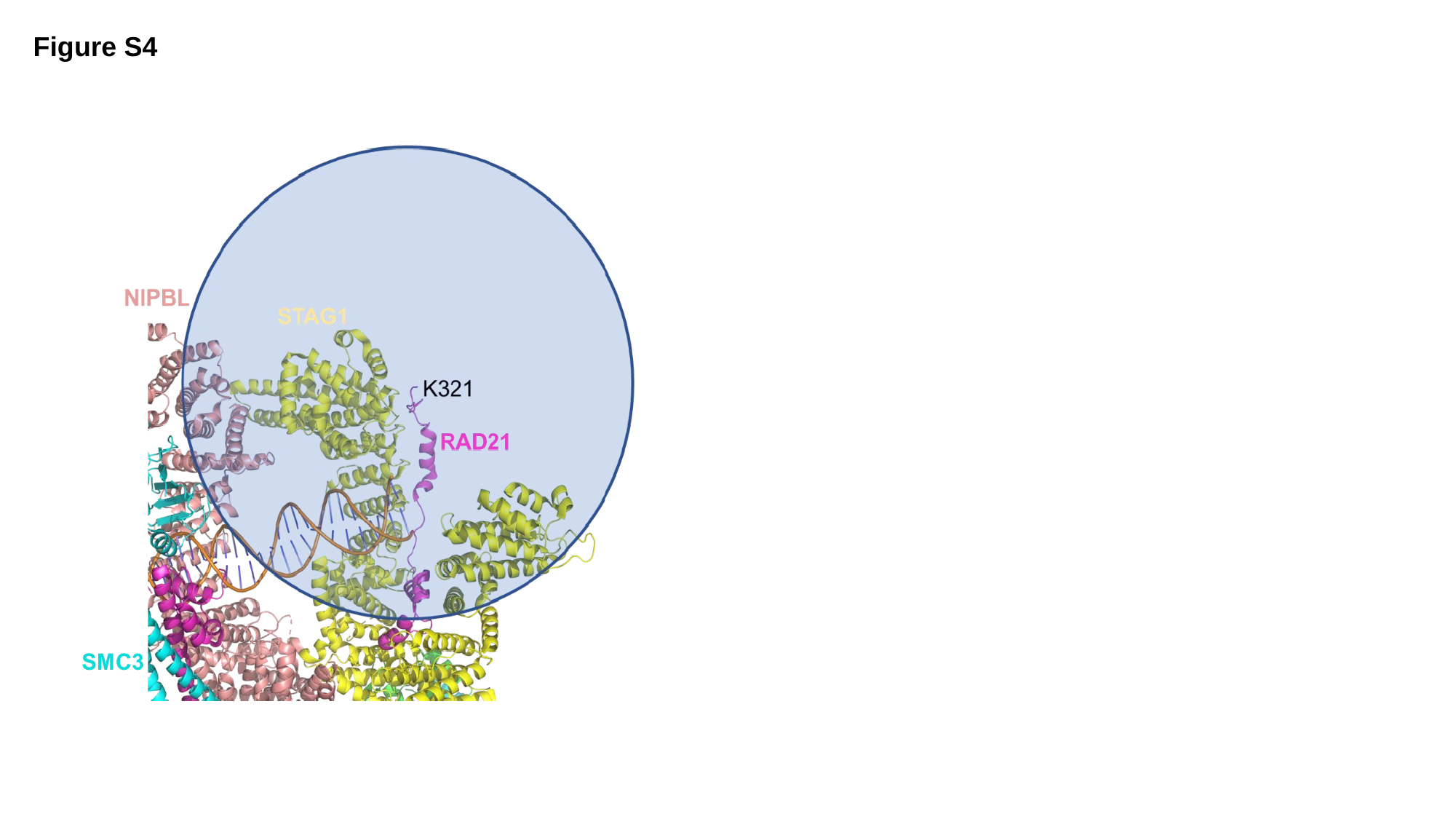

Figure S4

## Slide 5
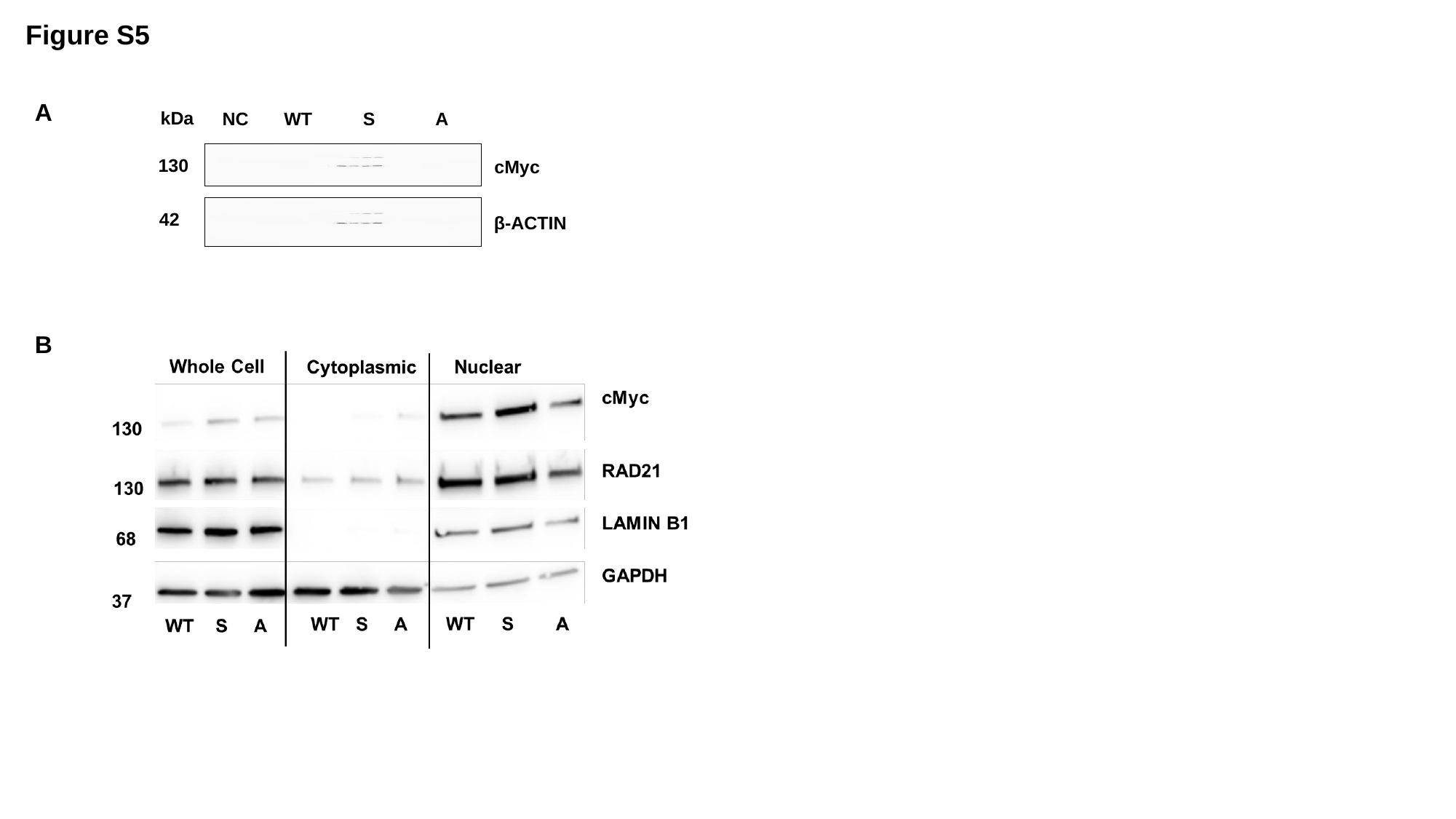

Figure S5
A
 NC WT S A
cMyc
130
β-ACTIN
 42
kDa
B

## Slide 6
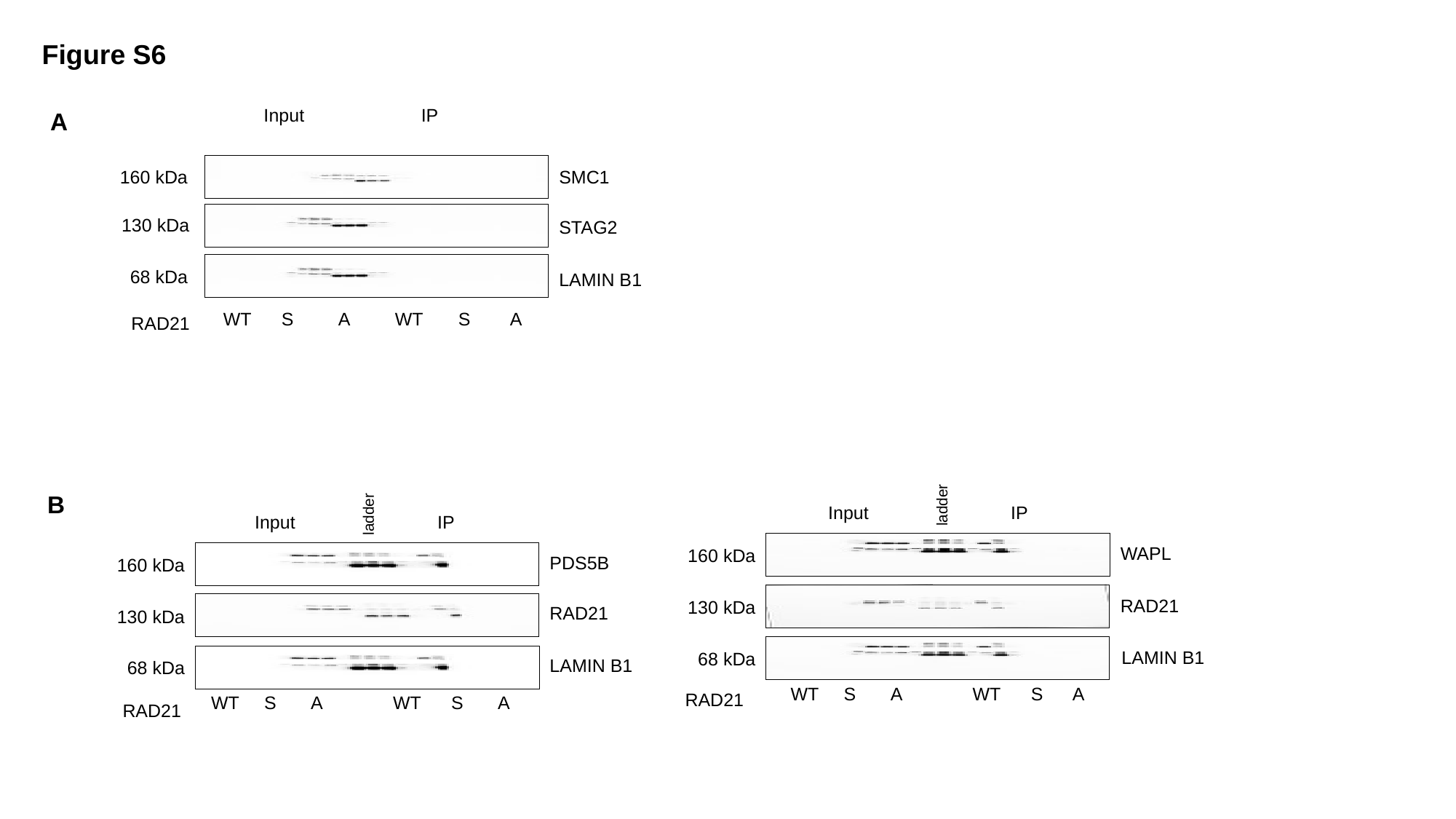

Figure S6
 Input IP
160 kDa
SMC1
130 kDa
STAG2
 68 kDa
LAMIN B1
WT S A WT S A
RAD21
A
ladder
 Input IP
WAPL
RAD21
LAMIN B1
 WT S A WT S A
ladder
B
 Input IP
160 kDa
PDS5B
160 kDa
130 kDa
RAD21
130 kDa
 68 kDa
LAMIN B1
 68 kDa
RAD21
RAD21
 WT S A WT S A

## Slide 7
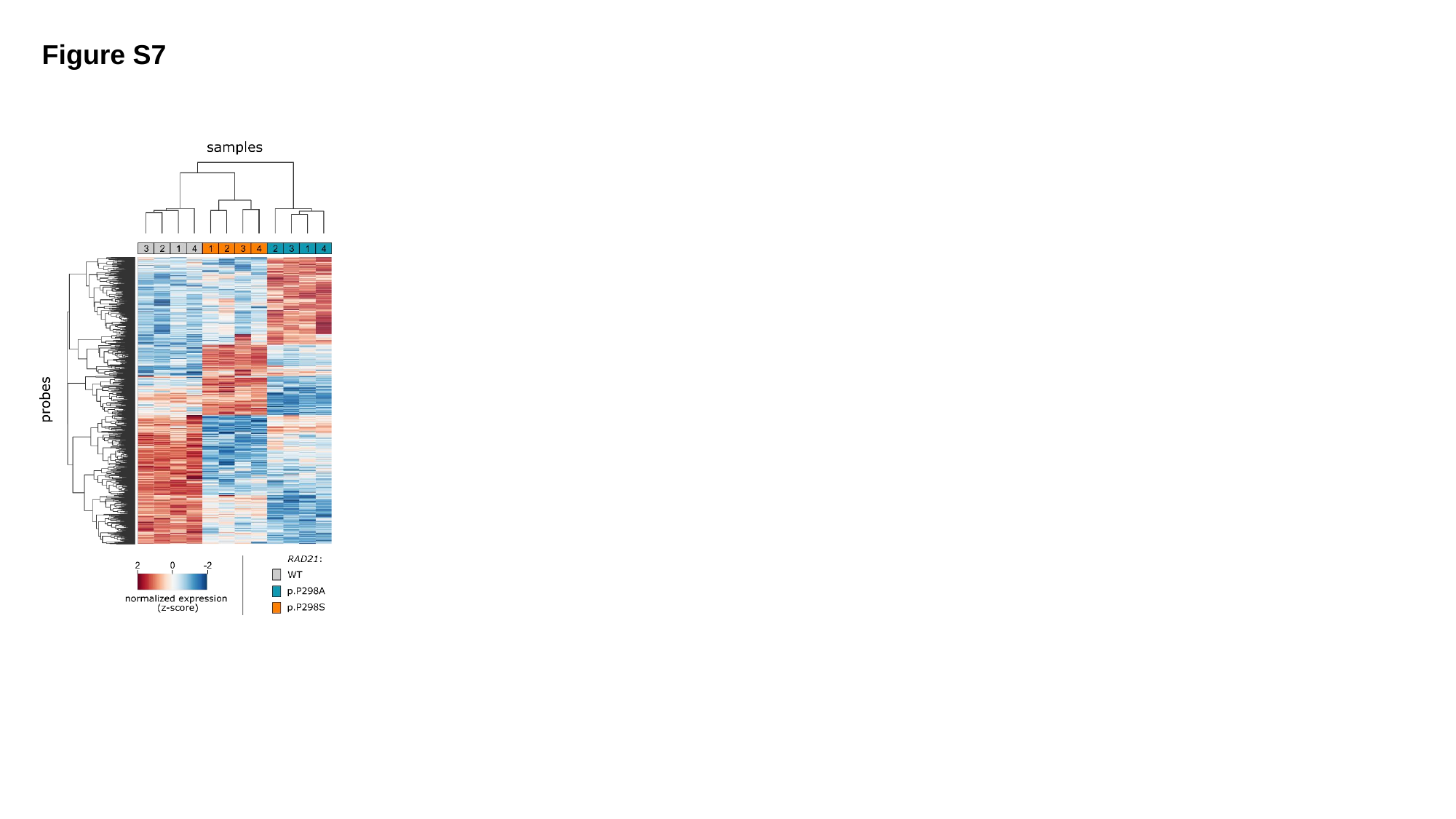

Figure S7

## Slide 8
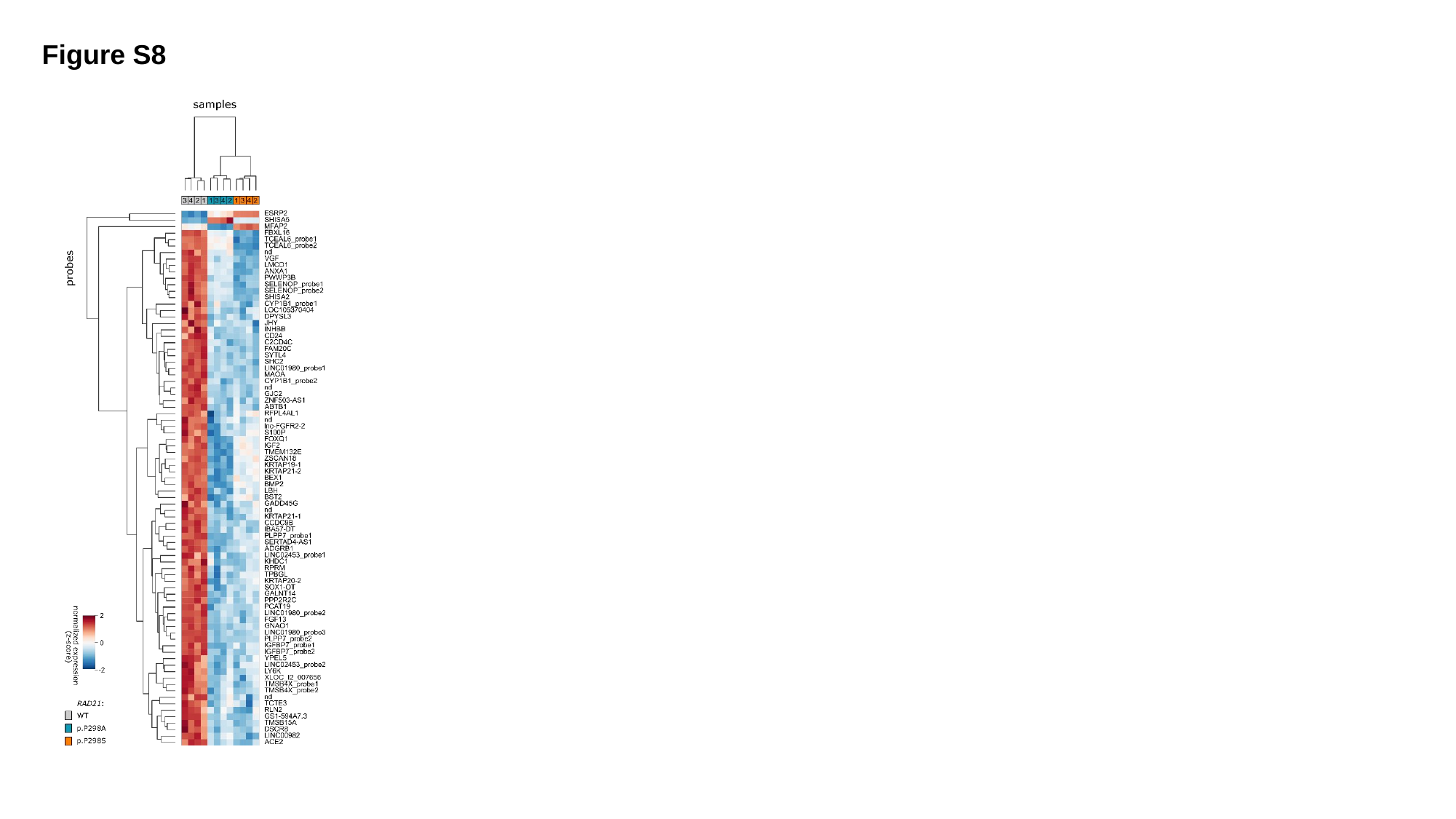

Figure S8

## Slide 9
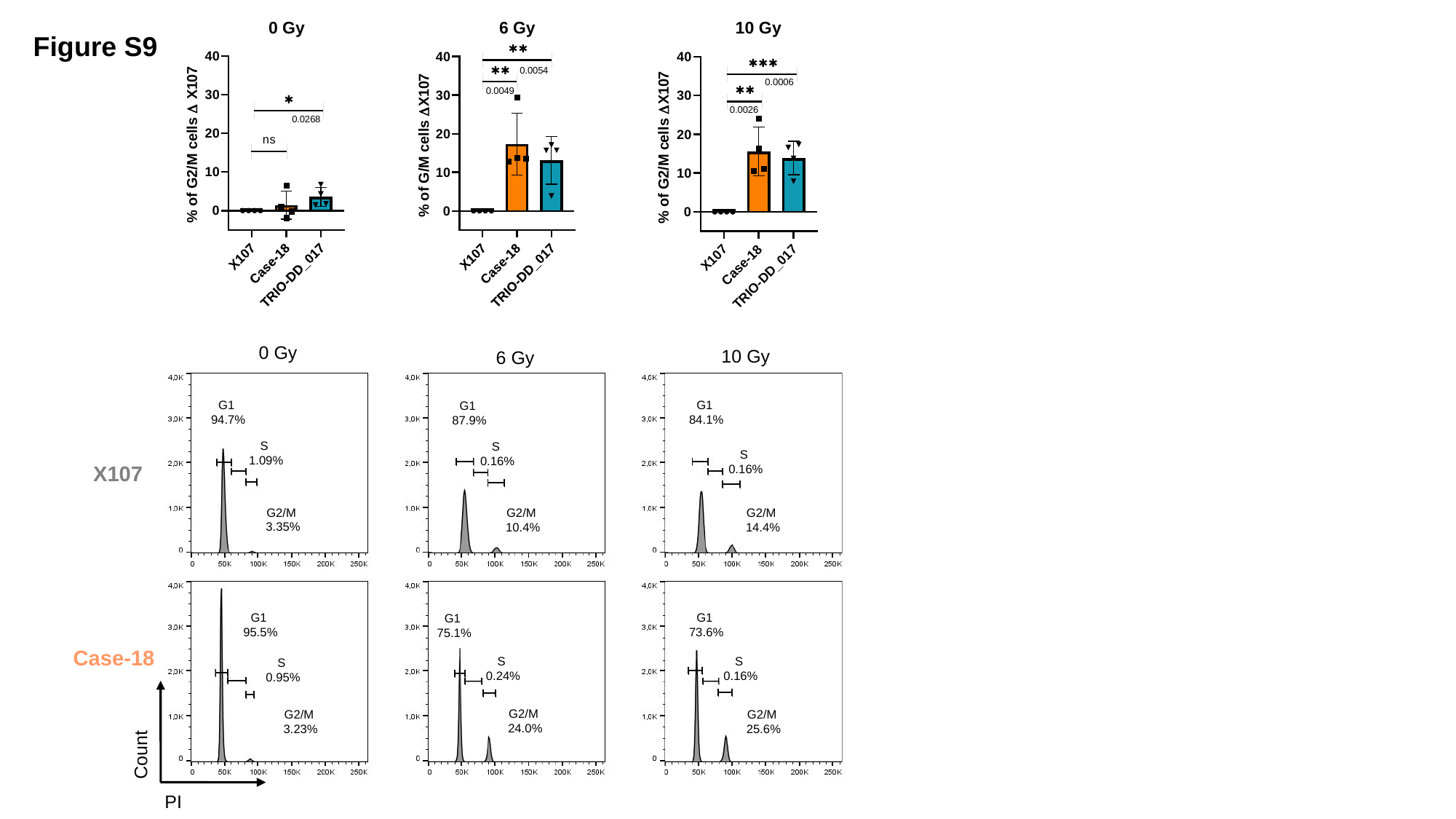

Figure S9
0 Gy
10 Gy
6 Gy
G1
94.7%
G1
84.1%
G1
87.9%
S
1.09%
S
0.16%
S
0.16%
X107
G2/M
3.35%
G2/M
10.4%
G2/M
14.4%
G1
73.6%
G1
95.5%
G1
75.1%
Case-18
S
0.16%
S
0.24%
S
0.95%
Count
PI
G2/M
24.0%
G2/M
25.6%
G2/M
3.23%

## Slide 10
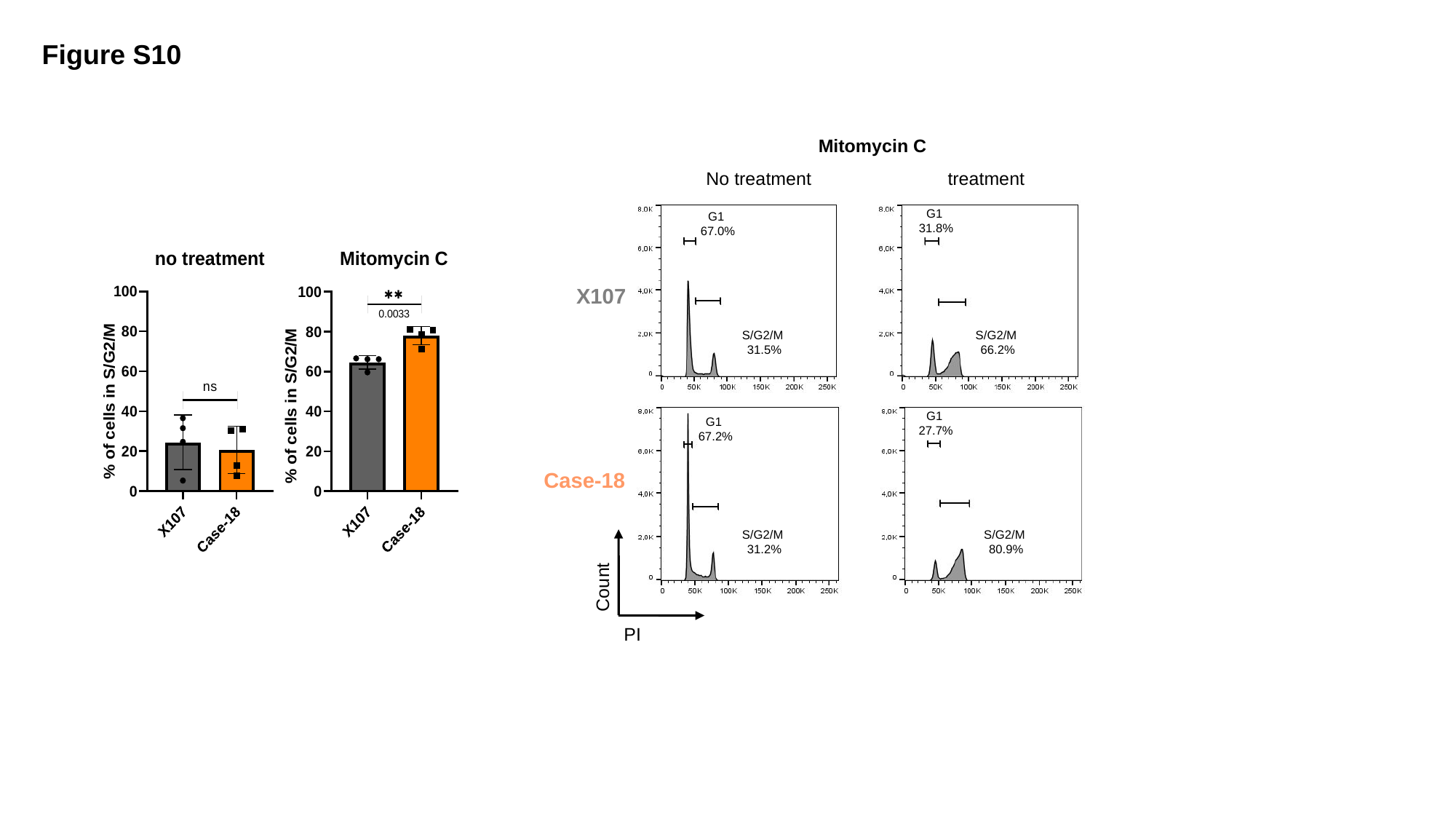

Figure S10
Mitomycin C
No treatment
treatment
G1
31.8%
G1
67.0%
X107
S/G2/M
31.5%
S/G2/M
66.2%
G1
27.7%
G1
67.2%
Case-18
S/G2/M
31.2%
S/G2/M
80.9%
Count
PI

## Slide 11
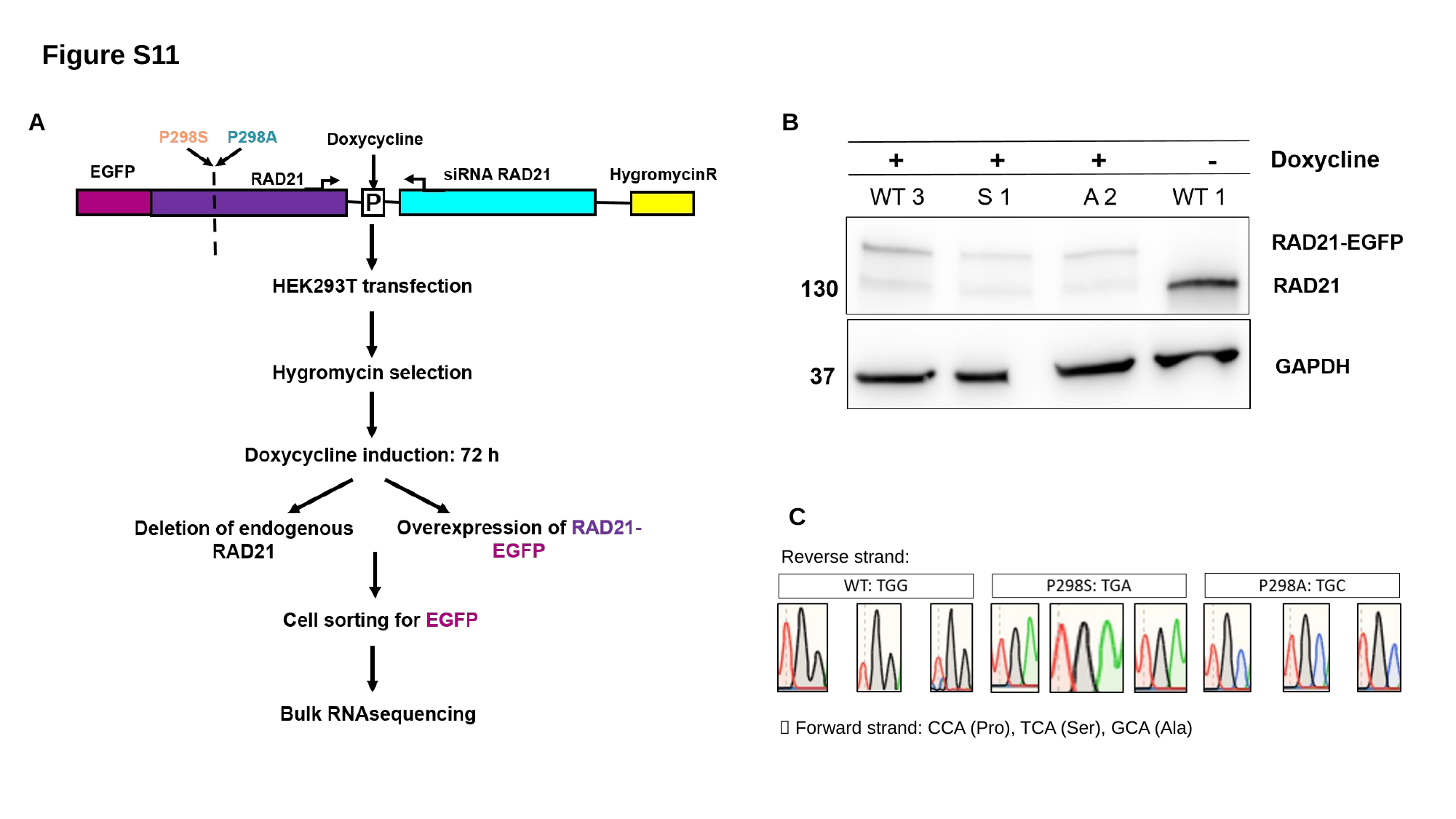

Figure S11
A
B
C
Reverse strand:
 Forward strand: CCA (Pro), TCA (Ser), GCA (Ala)

## Slide 12
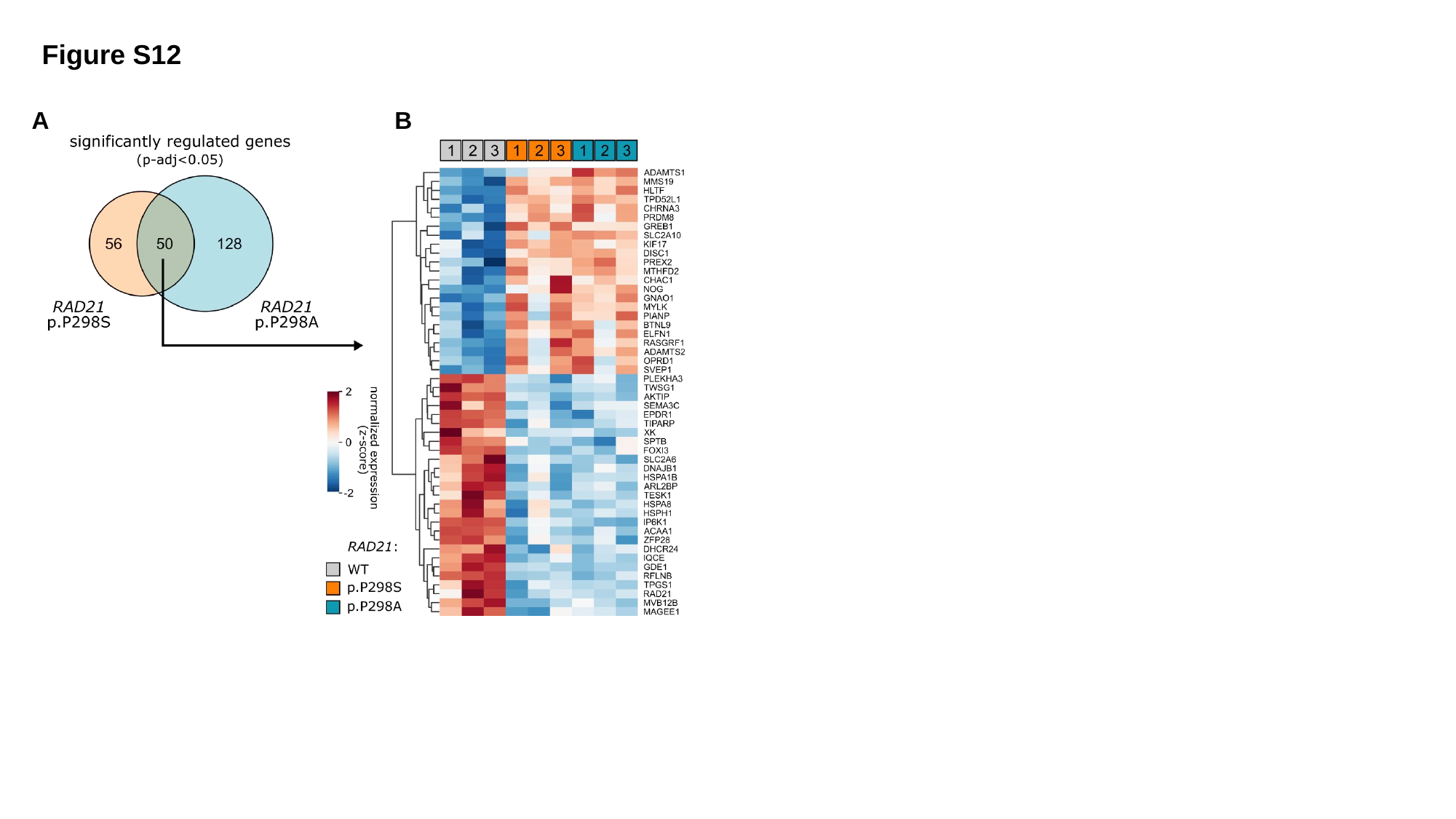

Figure S12
A
B

## Slide 13
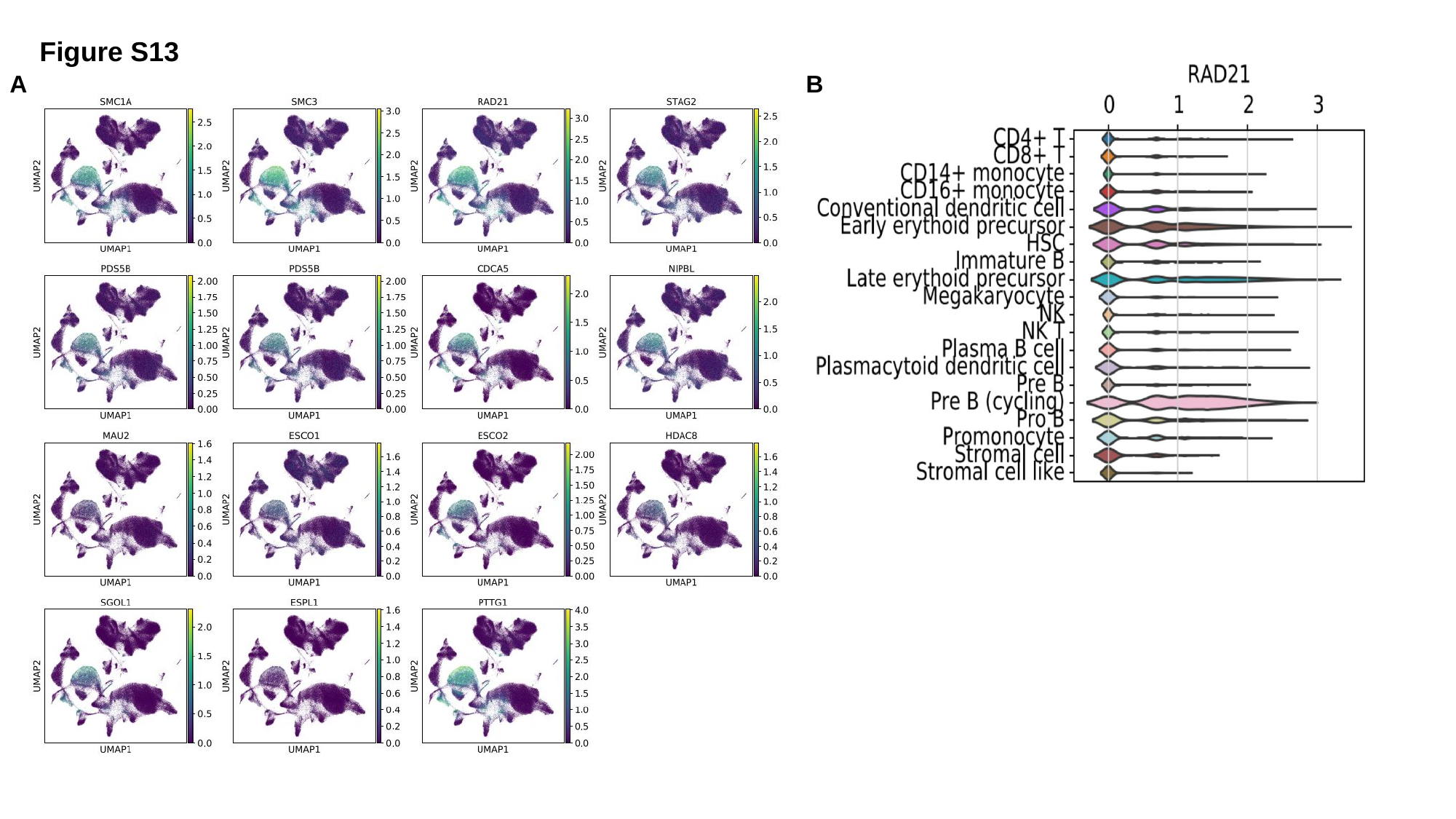

Figure S13
A
B

## Slide 14
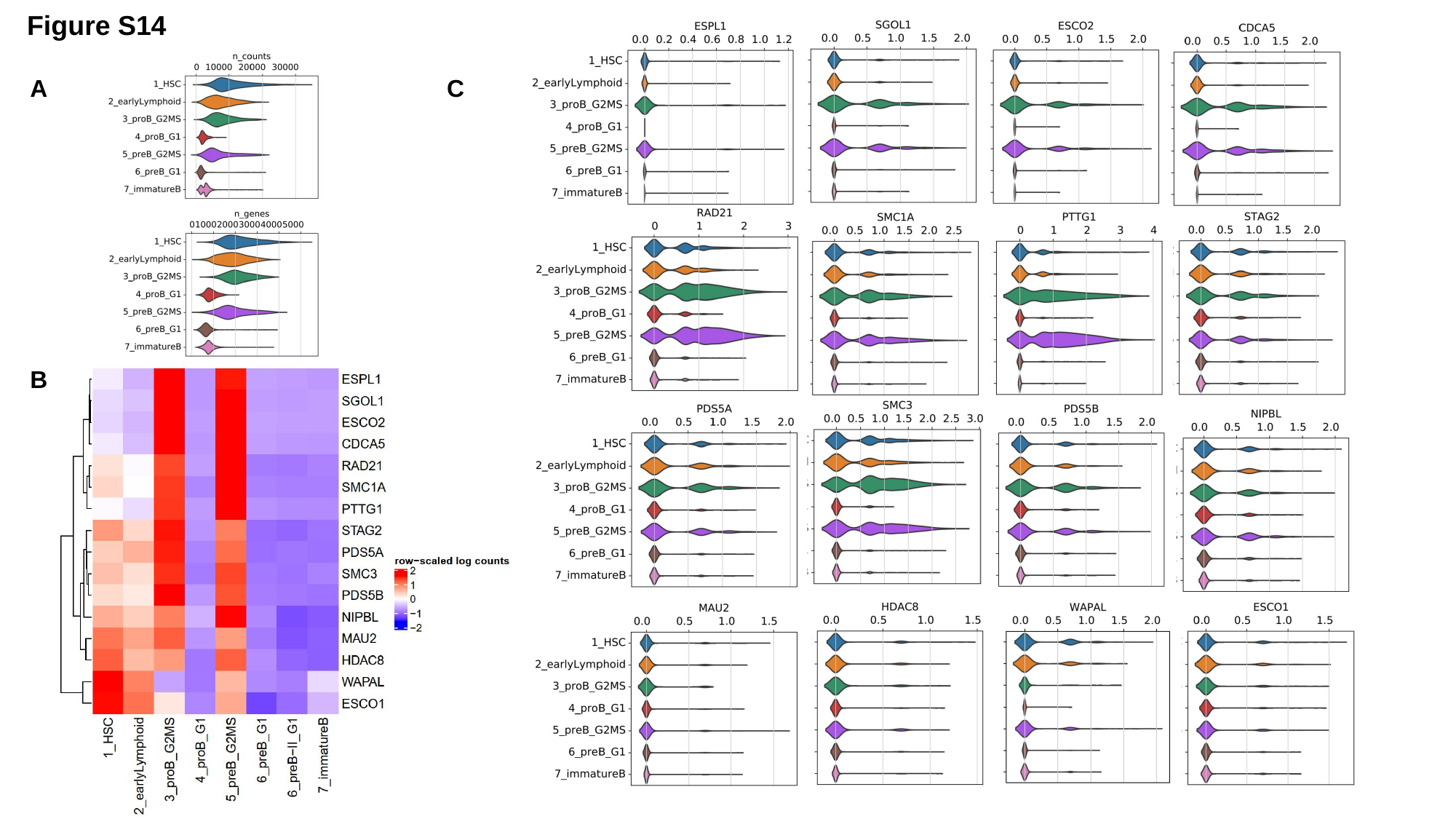

Figure S14
C
A
B

## Slide 15
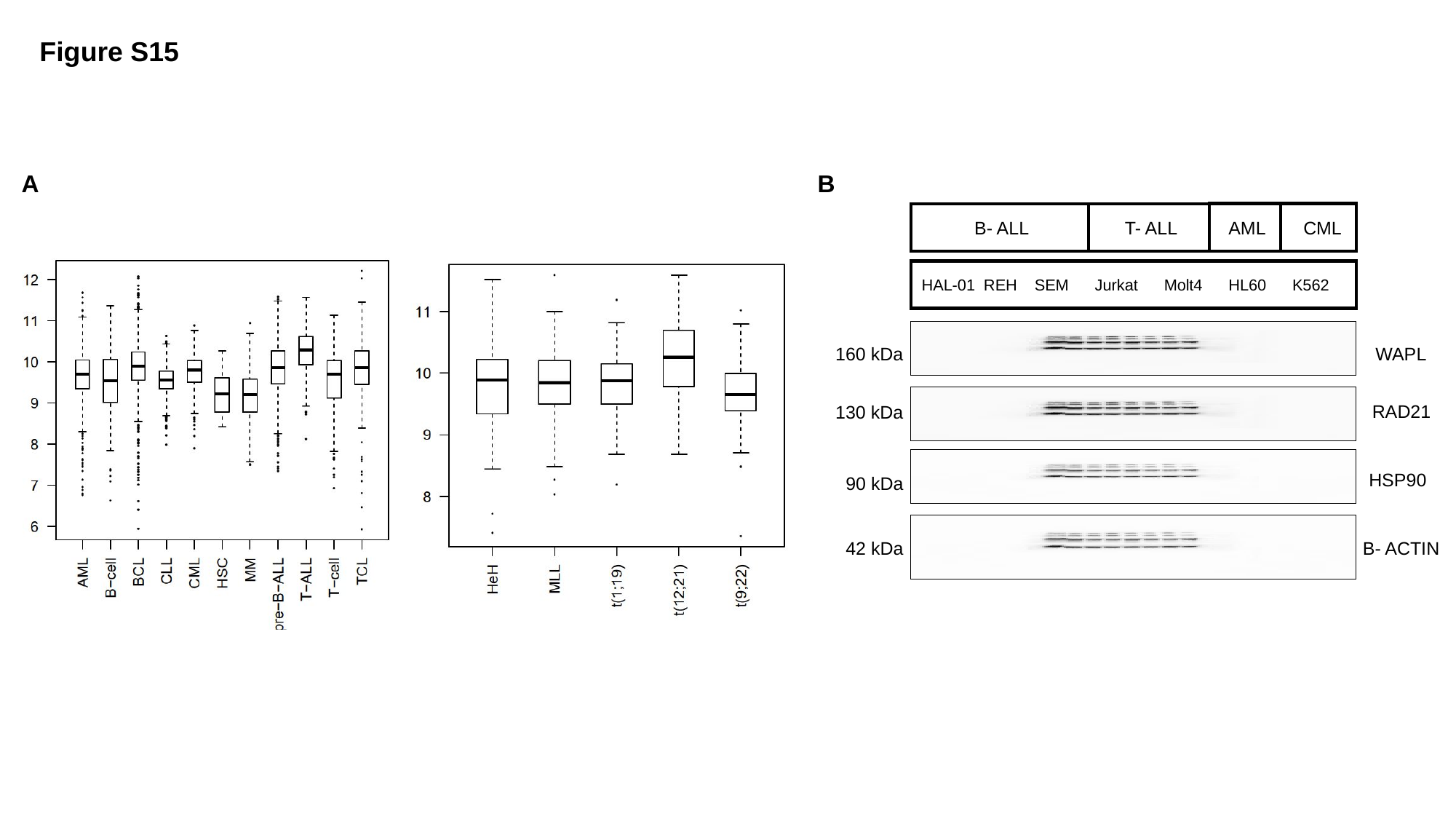

Figure S15
A
B
 CML
 AML
 B- ALL
 T- ALL
HAL-01 REH SEM Jurkat Molt4 HL60 K562
HSP90
WAPL
RAD21
B- ACTIN
160 kDa
130 kDa
 90 kDa
 42 kDa

## Slide 16
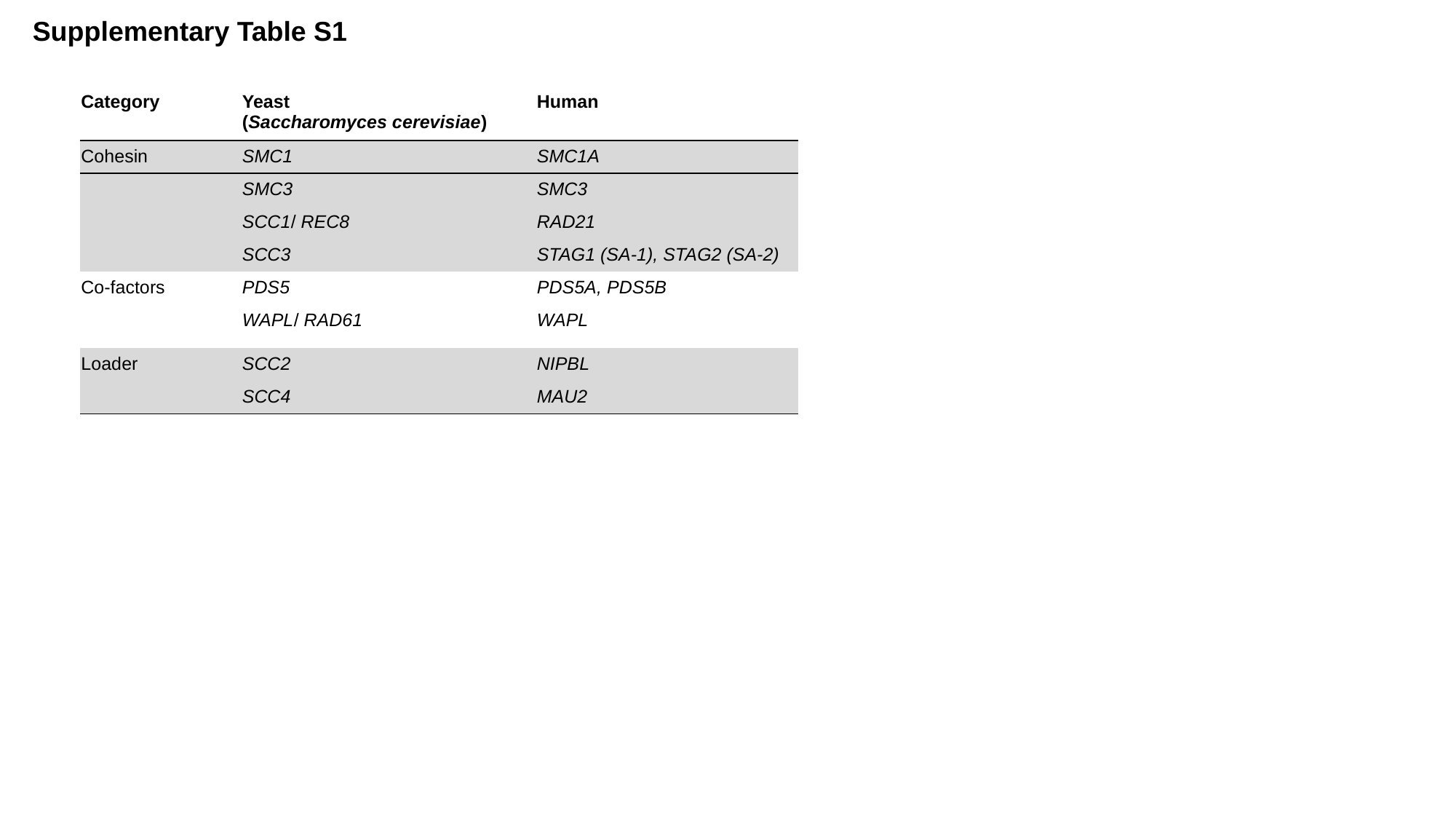

Supplementary Table S1
| Category | Yeast (Saccharomyces cerevisiae) | Human |
| --- | --- | --- |
| Cohesin | SMC1 | SMC1A |
| | SMC3 | SMC3 |
| | SCC1/ REC8 | RAD21 |
| | SCC3 | STAG1 (SA-1), STAG2 (SA-2) |
| Co-factors | PDS5 | PDS5A, PDS5B |
| | WAPL/ RAD61 | WAPL |
| Loader | SCC2 | NIPBL |
| | SCC4 | MAU2 |

## Slide 17
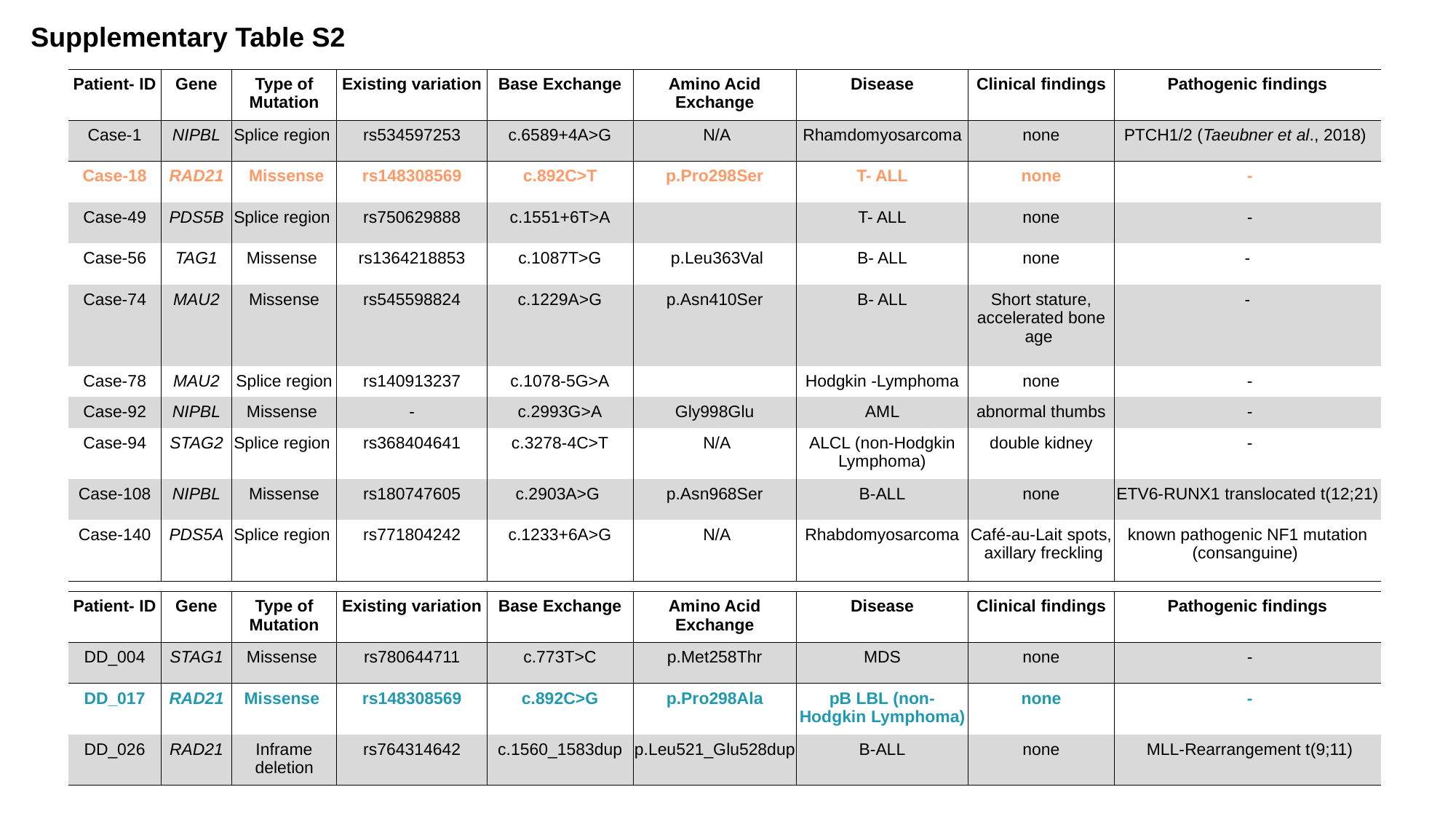

Supplementary Table S2
| Patient- ID | Gene | Type of Mutation | Existing variation | Base Exchange | Amino Acid Exchange | Disease | Clinical findings | Pathogenic findings |
| --- | --- | --- | --- | --- | --- | --- | --- | --- |
| Case-1 | NIPBL | Splice region | rs534597253 | c.6589+4A>G | N/A | Rhamdomyosarcoma | none | PTCH1/2 (Taeubner et al., 2018) |
| Case-18 | RAD21 | Missense | rs148308569 | c.892C>T | p.Pro298Ser | T- ALL | none | - |
| Case-49 | PDS5B | Splice region | rs750629888 | c.1551+6T>A | | T- ALL | none | - |
| Case-56 | TAG1 | Missense | rs1364218853 | c.1087T>G | p.Leu363Val | B- ALL | none | - |
| Case-74 | MAU2 | Missense | rs545598824 | c.1229A>G | p.Asn410Ser | B- ALL | Short stature, accelerated bone age | - |
| Case-78 | MAU2 | Splice region | rs140913237 | c.1078-5G>A | | Hodgkin -Lymphoma | none | - |
| Case-92 | NIPBL | Missense | - | c.2993G>A | Gly998Glu | AML | abnormal thumbs | - |
| Case-94 | STAG2 | Splice region | rs368404641 | c.3278-4C>T | N/A | ALCL (non-Hodgkin Lymphoma) | double kidney | - |
| Case-108 | NIPBL | Missense | rs180747605 | c.2903A>G | p.Asn968Ser | B-ALL | none | ETV6-RUNX1 translocated t(12;21) |
| Case-140 | PDS5A | Splice region | rs771804242 | c.1233+6A>G | N/A | Rhabdomyosarcoma | Café-au-Lait spots, axillary freckling | known pathogenic NF1 mutation (consanguine) |
| Patient- ID | Gene | Type of Mutation | Existing variation | Base Exchange | Amino Acid Exchange | Disease | Clinical findings | Pathogenic findings |
| --- | --- | --- | --- | --- | --- | --- | --- | --- |
| DD\_004 | STAG1 | Missense | rs780644711 | c.773T>C | p.Met258Thr | MDS | none | - |
| DD\_017 | RAD21 | Missense | rs148308569 | c.892C>G | p.Pro298Ala | pB LBL (non-Hodgkin Lymphoma) | none | - |
| DD\_026 | RAD21 | Inframe deletion | rs764314642 | c.1560\_1583dup | p.Leu521\_Glu528dup | B-ALL | none | MLL-Rearrangement t(9;11) |

## Slide 18
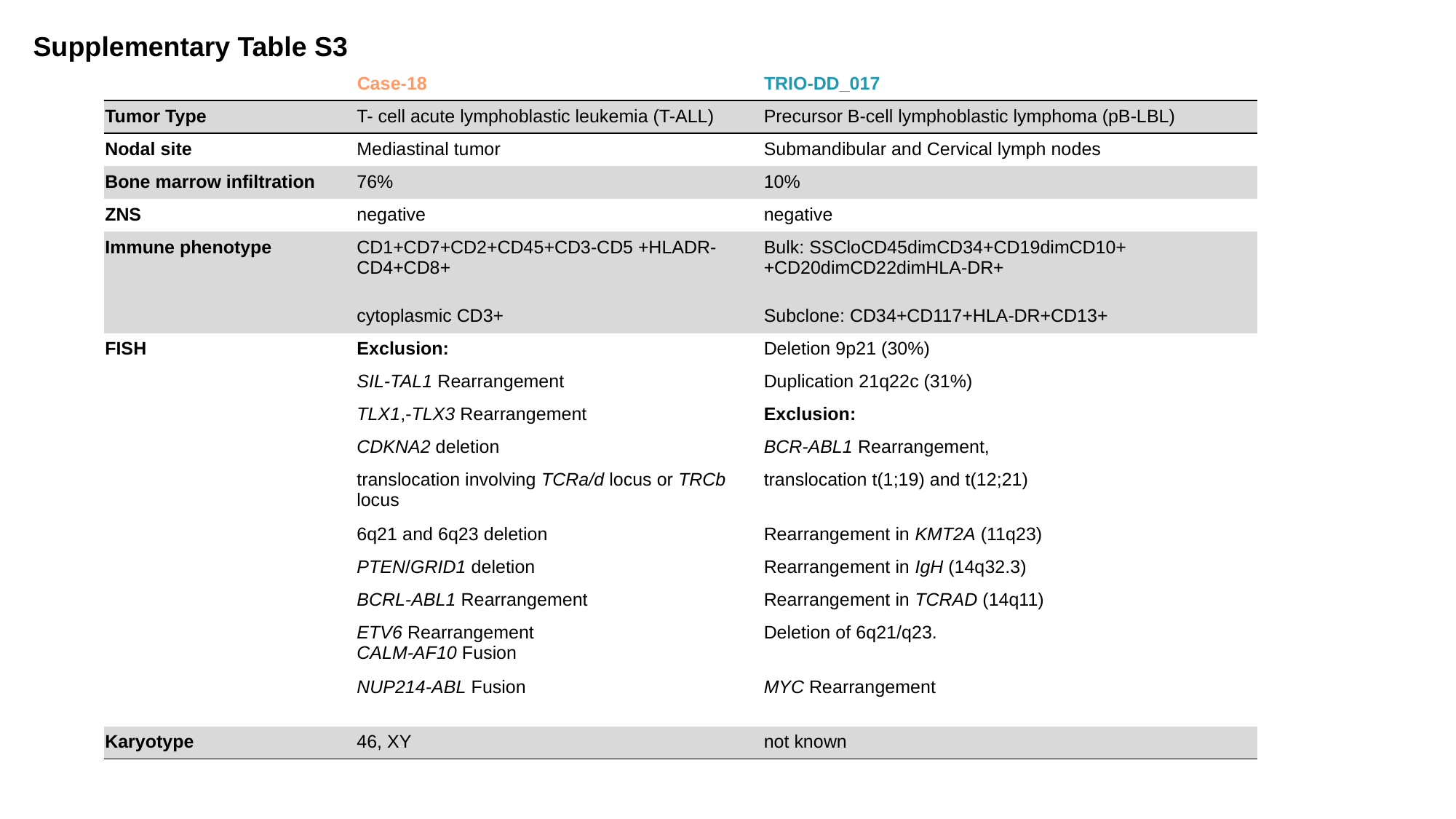

Supplementary Table S3
| | Case-18 | TRIO-DD\_017 |
| --- | --- | --- |
| Tumor Type | T- cell acute lymphoblastic leukemia (T-ALL) | Precursor B-cell lymphoblastic lymphoma (pB-LBL) |
| Nodal site | Mediastinal tumor | Submandibular and Cervical lymph nodes |
| Bone marrow infiltration | 76% | 10% |
| ZNS | negative | negative |
| Immune phenotype | CD1+CD7+CD2+CD45+CD3-CD5 +HLADR-CD4+CD8+ | Bulk: SSCloCD45dimCD34+CD19dimCD10++CD20dimCD22dimHLA-DR+ |
| | cytoplasmic CD3+ | Subclone: CD34+CD117+HLA-DR+CD13+ |
| FISH | Exclusion: | Deletion 9p21 (30%) |
| | SIL-TAL1 Rearrangement | Duplication 21q22c (31%) |
| | TLX1,-TLX3 Rearrangement | Exclusion: |
| | CDKNA2 deletion | BCR-ABL1 Rearrangement, |
| | translocation involving TCRa/d locus or TRCb locus | translocation t(1;19) and t(12;21) |
| | 6q21 and 6q23 deletion | Rearrangement in KMT2A (11q23) |
| | PTEN/GRID1 deletion | Rearrangement in IgH (14q32.3) |
| | BCRL-ABL1 Rearrangement | Rearrangement in TCRAD (14q11) |
| | ETV6 Rearrangement CALM-AF10 Fusion | Deletion of 6q21/q23. |
| | NUP214-ABL Fusion | MYC Rearrangement |
| Karyotype | 46, XY | not known |

## Slide 19
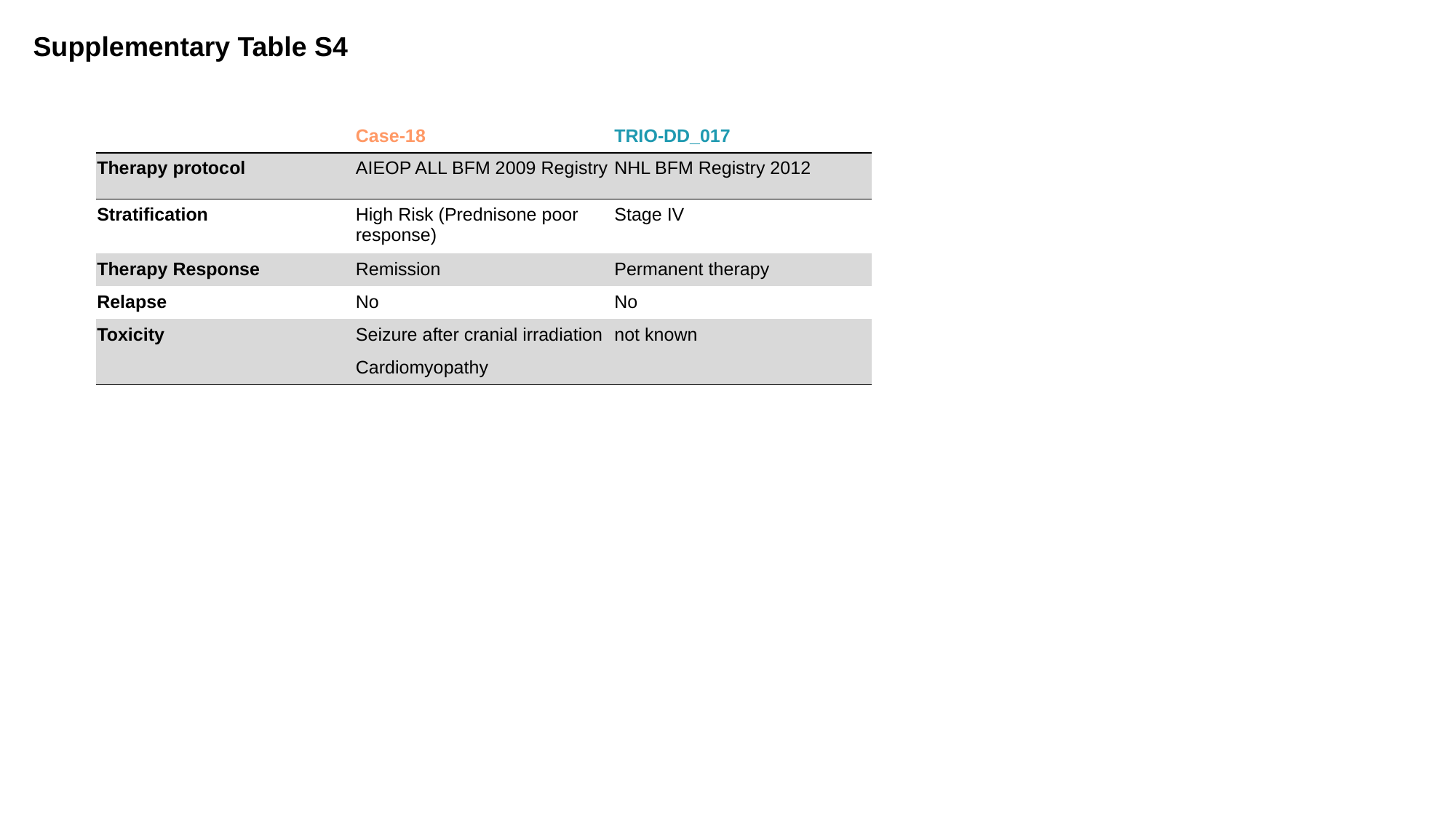

Supplementary Table S4
| | Case-18 | TRIO-DD\_017 |
| --- | --- | --- |
| Therapy protocol | AIEOP ALL BFM 2009 Registry | NHL BFM Registry 2012 |
| Stratification | High Risk (Prednisone poor response) | Stage IV |
| Therapy Response | Remission | Permanent therapy |
| Relapse | No | No |
| Toxicity | Seizure after cranial irradiation | not known |
| | Cardiomyopathy | |

## Slide 20
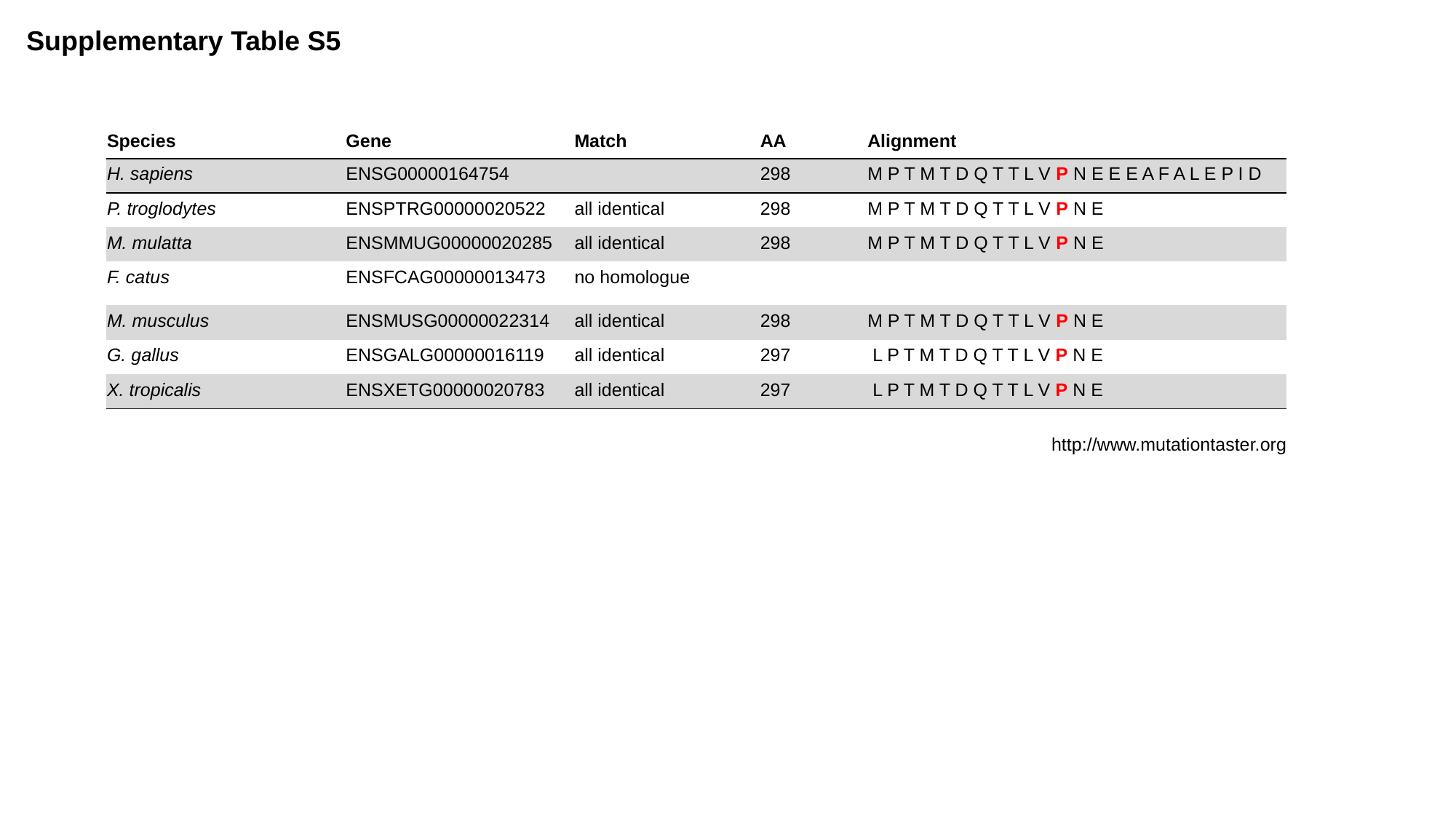

Supplementary Table S5
| Species | Gene | Match | AA | Alignment |
| --- | --- | --- | --- | --- |
| H. sapiens | ENSG00000164754 | | 298 | M P T M T D Q T T L V P N E E E A F A L E P I D |
| P. troglodytes | ENSPTRG00000020522 | all identical | 298 | M P T M T D Q T T L V P N E |
| M. mulatta | ENSMMUG00000020285 | all identical | 298 | M P T M T D Q T T L V P N E |
| F. catus | ENSFCAG00000013473 | no homologue | | |
| M. musculus | ENSMUSG00000022314 | all identical | 298 | M P T M T D Q T T L V P N E |
| G. gallus | ENSGALG00000016119 | all identical | 297 | L P T M T D Q T T L V P N E |
| X. tropicalis | ENSXETG00000020783 | all identical | 297 | L P T M T D Q T T L V P N E |
http://www.mutationtaster.org
