# Supplementary material for: Recurrent Germline Variant in RAD21 Predisposes Children to Lymphoblastic Leukemia or Lymphoma
Source: Int J Mol Sci. 2022 May 5;23(9):5174. doi: 10.3390/ijms23095174 (PMC9106003; doi:10.3390/ijms23095174)
Supplement: Supplementary file 1 [file ijms-23-05174-s001.zip › ijms-1701368-supplementary/Schedel et al_Supplementary Information_final.pdf]

Supplementary Information (*Schedel et. al.*)

Supplementary Figure Legends:

Supplementary Figure S1: Coding variants in cohesin complex genes identified per patient

Distribution of coding variants (MAF <0.1%) in cohesin genes among patients illustrating that all cohesin variants are mutually exclusive.

Supplementary Figure S2: Genomic validation of *RAD21* p.P298S/A

Sanger-Sequencing for *RAD21* p.P298S/A variant confirmation was performed for Case-18 and TRIO-DD_017.

Supplementary Figure S3: Analysis of pathogenic variants in patients carrying *RAD21* p.P298S/A

Analysis of Case-18 and TRIO-DD_017 variants by applying the Cancer Predisposition Sequencing Reporter (CPSR) algorithm. No pathogenic or likely pathogenic variants were found within the complete dataset.

Supplementary Figure S4: *In-silico* modeling of *RAD21* p.P298S/A

3D crystal structure of RAD21, showing putative interaction partners of P298 and the substitutions P298S and P298A. The possible range in which residue 298 can move is shown as a blue sphere centered on K321, the nearest, structurally resolved residue in the cryo-EM structure 6WG3. Putative interaction partners include residues in STAG1, NIPBL, SMC3, and the double-stranded DNA.

Supplementary Figure S5: Gene expression and nuclear export are not altered through *RAD21* p.P298S/A

A: Immunoblot of HEK293T cells, which were transfected with cMyc-tagged RAD21 Wildtype (WT), RAD21 p.P298S (S) or RAD21 p.P298A (A) after colchicine arrest.

B: Immunoblotting of whole-cell lysates and cytoplasmic and nuclear extracts was performed in transfected HEK293T cells, confirming that the cytoplasmic-nuclear localization is not affected by either RAD21 P298S or P298A. Lamin B1 serves as nuclear and GAPDH as cytoplasmic control.

Supplementary Figure S6: Cohesin complex formation is not disturbed by *RAD21* p.P298S/A

A: Immunoblot of immunoprecipitation displayed in **Figure 2A** with cMyc-tag blotted with other cohesin components (SMC1 and SA2 (=STAG2)).

B: Immunoblot of immunoprecipitation performed with RAD21 as the target instead of cMyc-tag as described in **A**.

Supplementary Figure S7: Gene expression profiles of *RAD21* p.P298S/A

Two-dimensional hierarchical clustering and heat map representation of microarray data: Gene expression of HEK293T cells overexpressing R32-hRAD21 WT, p.P298S or p.P298A is analyzed in four replicates, each. Dendrograms represent the clustering of samples (top) and gene probes (left). Sample description is indicated by colors and replicate number below the sample dendrogram at the top of the heat map. Normalized expression values of all differentially expressed (>50% up-/downregulation, adjusted p-value <0.05) gene probes (n=995) are z-transformed and hierarchically clustered (Euclidean distance, complete linkage) for probes and samples. All samples are included (n=12).

Supplementary Figure S8: Differentially regulated genes overlapping for *RAD21* p.P298S and *RAD21* p.P298A

Two-dimensional hierarchical clustering and heat map representation of microarray data restricted to probes that are differentially expressed (|fc| >50% and adj. p-value <0.05) in both *RAD21* variants as represented in **Figure 2B**. Analysis and representation as in **Supplementary Figure 7**.

Supplementary Figure S9: Cell cycle arrest in primary patient fibroblasts after irradiation

Upper: Results of cell cycle analysis of X107, Case-18, and TRIO-DD_017 primary fibroblasts after being subjected to irradiation with 6 and 10 Gy, respectively (n=4). Student's t-testing was performed, with indicated p-values. Case-18 and TRIO-DD_017 were adjusted to X107 as a baseline response.

Lower: representative FACS data image and gating.

Supplementary Figure S10: Cell cycle arrest in primary patient fibroblasts after Mitomycin-C treatment

Cell cycle analysis of primary fibroblasts of X107 and Case-18, which were further subjected to Mitomycin-C treatment (n=4). Cells were either left untreated or treated for 5 min with a concentration of 0.1 mg/ml of Mitomycin-C 24 h before analysis. Indicated p-values were calculated by Student's t-test. Left: Summary of four replicates. Right: representative FACS Data image and gating.

Supplementary Figure S11: Inducible expression of RAD21 WT and variants with concomitant downregulation of endogenous RAD21

A: Inducible System (pRTS-1-RAD21) and work flow scheme

B Western Blot validation of *RAD21* WT vs. p.P298S and p.P298A, 72 h after Doxycycline induction and cell sorting for EGFP.

C: Transcriptional validation of *RAD21* WT vs. p.P298S and p.P298A (3 transfections each) after Doxycycline induction (reverse strand).

Supplementary Figure S12: Differentially expressed genes compared between *RAD21* p.P298S and *RAD21* p.P298A (pRTS-1-RAD21)

A: Gene expression is measured by bulk RNA-Sequencing of *RAD21* WT, p.P298S and p.P298A in three biological replicates – see **Figure 3A**. Fold-changes in gene expression is calculated between *RAD21* p.P298S vs. WT and *RAD21* p.P298A vs. WT. Significantly regulated genes are defined by an up- or down-regulation with an adjusted p-value <0.05.

B: One-dimensional hierarchical clustering and heat map representation of bulk RNA-Sequencing data from **A** restricted to genes that are differentially expressed (adj. p-value <0.05) in both *RAD21* variants compared to the WT. Samples are indicated as colors and replicate numbers above the heat map and are not automatically clustered. Normalized count values for all 50 included genes are z-transformed and hierarchically clustered (Euclidean distance, complete linkage).

Supplementary Figure S13: Single-cell RNA-Sequencing data depicting cohesin expression in the human bone marrow

A: UMAP-visualizations showing the expression levels of cohesin complex genes in individual cells.

B: Violin plot showing RAD21 expression in different cells in healthy bone marrow scRNA-Seq data.

Supplementary Figure S14: Single-cell RNA-Sequencing data depicting cohesin expression during B-cell differentiation in humans

A: Violin plots showing the number of counts and genes detected in cells of the B-cell differentiation stated from healthy bone marrow scRNA-Seq data.

B: A row-scaled heatmap showing the expression of the cohesin complex genes in the different stages of B-cell differentiation.

C: Violin plots showing the expression level of cohesin genes in the different states of the B-cell differentiation.

Supplementary Figure S15: RAD21 expression in leukemia

A: Normalized gene expression from several hematological malignancies and healthy cell types (left), and subtypes of pre-B-ALL (right) from the Hemap dataset (http://doi.org/10.1158/0008-5472.CAN-18-2970). AML: Acute Myeloid Leukemia. B-cell: healthy B-cell. BCL: B-cell Lymphoma. CLL: Chronic Lymphoblastic Leukemia. CML: Chronic Myeloid Leukemia. HSC: Hematopoietic Stem Cell. MM: Multiple Myeloma. Pre-B-ALL: Pre-B-cell Acute Lymphoblastic

Leukemia. T-ALL: T-cell Acute Lymphoblastic Leukemia. T-cell: healthy T-cell: TCL: T-cell Lymphoma. HeH: hyperdiploid. MLL: MLL-rearranged. t(1;19): TCF3-PBX1. t(12;21): ETV6-RUNX1. t(9;22): BCR-ABL1.

B: Protein expression of WAPL and RAD21 among various B-ALL, T-ALL and AML cell lines, showing that RAD21 and WAPL are expressed ubiquitously.

Supplementary Table S1: Cohesin complex genes

Gene panel of the cohesin complex genes, adapted from *Losada* et al, 2016.

Supplementary Table S2: Patients harboring cohesin gene variants

Genetic, clinical and phenotypical findings which emphasize on possible CdLS phenotype of the patients with cohesin complex germline variants (MAF <0.1%).

Supplementary Table S3: Presentation of leukemia and lymphoma in index patients

Immuno- and molecular genetic tumor analyses for Case-18 and TRIO-DD_017 performed at diagnosis.

Supplementary Table S4: Clinical characteristics of index patients

Treatment history of Case-18 and TRIO-DD_017.

Supplementary Table S5: Conservation of amino acid 298 in *RAD21*

RAD21 p.P298 is conserved among species. Data was adapted from http://www.mutationstaster.org/

Supplementary Table S6: Gene expression data of the 83 overlapping genes

Table of genes significantly up-/down-regulated in *RAD21* p.P298A/S compared to the WT in the R32-hRAD21 cell system.

Supplementary Table S7: Gene expression data of the 50 overlapping genes

Table of genes significantly up-/down-regulated in *RAD21* p.P298A/S compared to the WT in the inducible pRTS-1-RAD21 cell system.

Supplementary Material & Methods:

Whole exome sequencing (WES):

In short, after the generation of read files in fastq format using bcl2fastq v2.19.0, trimmomatic v0.33 was used to remove adapter and low-quality sequences (1). The alignment to the human reference genome GRCh37 was performed using BWA-MEM v0.7.12 (2) and Samtools v1.2 (3). The tool Peddy 0.4.6 (4) performed gender and relatedness analyses to validate the correct sample assignment and the expected relationship of the patient's data with the corresponding parents' data. Single nucleotide variants (SNVs) and insertion/deletions (indels) were called using GATK v4.1.4.1 and VarScan2 v2.3.9 (5), applying the trio-mode. Functional annotation of variants was done using Ensembl Variant Effect Predictor v98.3 (6). For in silico prediction of the effect of the variants, SIFT, Polyphen and CADD were applied. The COSMIC database (downloaded 25.03.2019 https.//cancer.sanger.ac.uk/cosmic/download) was used to identify variants located in somatic mutational hotspots. In addition, we used the ClinVar database (download 02/12/2019), the IARC TP53 germline database and the LOVD database for MSH2, MSH6, APC and NF1 in order to identify previously reported pathogenic variants. Furthermore, we used the dbNSFP 3.5 plugin to annotate the conservation scores based on GERP++ and phastCons100way_vertebrate. For in silico prediction of the effect of splice site variants, we applied the dbscSNV v1.1 plugin for VEP (7), which annotated

the ada- and rf-scores to the splice variants.

The initial variant interpretation was carried out with the CPSR pipeline (8), which classified the variants as pathogenic, likely pathogenic, variant of unknown significance (VUS), likely benign, or benign. The additional variant interpretation was manually performed (e.g. by taking CADD scores into account (9)) as well as by utilizing an extended cancer gene list.

Sanger Sequencing Validation:

RAD21 p.P298S and *RAD21* p.P298A were validated via PCR and subsequent Sanger sequencing using the following primers (5′ 3′):

**Name**	**Sequence (5‘ 3‘)**	**Patient**
hRAD21_Ex8_F	ATTGGGTTCAAGTCTGGCGG	Case-18
hRAD21_Ex8_R	TTCTGGGAACCCCAGGAGAC	Case-18
hRAD21_Ex8_F	ATTGGGTTCAAGTCTGGCGG	TRIO-DD_017
hRAD21_Ex8_R	TTCTGGGAACCCCAGGAGAC	TRIO-DD_017

***RAD21* Variation Analysis:**

RAD21, transcript ID ENST00000297338 was analyzed. Minor allele frequencies of all coding germline variants present in *RAD21* in a global, non-cancer population, taken from the gnomAD exome r.2.1.1 dataset (n=118.479), were summed up codon-wise. The variants had to be VEP annotated to one of the following consequences for inclusion: start_lost, missense_variant, inframe_insertion, inframe_deletion, stop_gained,

frameshift_variant, coding_sequence_variant, stop_lost, incomplete_terminal_codon_variant, transcript_ablation, transcript_amplification, protein_altering_variant. Somatic, coding variants reported for adult cancer patients derive from COSMIC, GRCh37 Release 91 (CosmicCodingMuts.normal.vcf.gz, n=1,443,198 samples) and were similarly combined for each codon along *RAD21*. Both collected datasets were smoothed using the LOWESS algorithm (fraction: 0.06, iterations: 3) prior to plotting.

***In-silico* Modeling:**

To assess the structural impact of the P298S and P298A substitutions in RAD21, we aimed at structurally modeling the 50 residues on each side of the substitutions. However, several homology modeling and *ab initio* modeling approaches failed to generate a secondary structure for this region. This reflects that the substitution site is part of a very flexible and likely intrinsically disordered region. MFDp2 (10), a disorder predictor, also predicts this part to be disordered, (predicted disorder content of the entire RAD21: 51.7%). Accordingly, a recent cryo-electron microscopy (cryo-EM) structure contains the largest structurally resolved part of RAD21 in addition to (partially) resolved binding partners STAG1, SMC1, SMC3, and NIPBL and double-stranded DNA (PDB ID: 6WG3) (11). However, the part of RAD21 containing the substitution site was not resolved because of the high flexibility of this region. From the

cryo-EM structure, estimating with which residues P298 and the substitutions P298S and P298A may interact results in many putative interaction partners in STAG1, SMC3, NIPBL and the double-stranded DNA. Hence, we aimed at narrowing down putative interaction partners based on sequence analyses. We used the HMMER suite (12) to produce a multiple sequence alignment, applying jackhammer and using an E-value cut-off of 10-6 and minimum coverage of 75%. The alignment reveals that proline and serine can occur at amino acid 298. Alanine also occurs at this position, but with a lower probability than proline or serine. Next, a possible co-evolution of P298 within RAD21 was determined via GREMLIN (13), using the full-length sequence and, first, default parameters as well as, second, an E-value cut-off of 10^{-6}, minimum coverage of 25%, and gap removal of 50% using HHblits. Both searches did not leave enough sequences to generate a co-evolution analysis.

Immunoblotting:

For whole-cell lysates, $7x10^6$ HEK293T cells stably overexpressing hR32-hRAD21 WT, p.P298S or p.P298A were lysed in RIPA Buffer (50 mM TRIS, 150 mM NaCl, 0.5% Sodium Deoxycholate, 1% TRITON and 0.1% SDS 20%, with 10x PhosSTOP (PS, Roche) and 25x PIC (Protease Inhibitor Cocktail, Roche) freshly added), for 30 min on ice, while vortexing every 5 min. Whole-cell lysates of leukemia cells (8-10 x 10^6 cells) were lysed equally (3 biological replicates). For MycTaq validation HEK293T, HEK293T stably

overexpressing R32-hRAD21 WT, HEK293T R32-hRAD21 p.P298S and HEK293T R32-hRAD21 p.P298A were arrested with colchicine as described in "Immunoprecipitation" and lysed with 10 mM TRIS-HCl (pH 8.0), 1 mM EDTA, 100 mM NaCl, 2 mM $MgCl_2$, 10% NP40, 25x PIC and 10x PS kept 30 min on ice with vortexing every 5 min. Protein concentration was measured with the Bradford protein assay (Roti-Quant, Roth) by determining the OD_{595nm}. Cytoplasmic and nuclear lysates were prepared from $10x10^6$ cells using NE-PER Nuclear and Cytoplasmic Extraction Reagents (Thermofisher, adapted for $10x10^6$ cells). 12 μg (for HEK293T cells samples) or 15 ug (for leukemia cell samples and MycTaq validation blot) were heated for 10 min at 95 °C while shaking at 350 rpm and loaded accordingly onto BIORAD Mini-Protean TGX Gel 4-20%. The blot was run cold (only HEK293T samples) for 20 min at 70 V, following ~90 min at 120 or 130 V. Transfer was performed using the Trans-Blot Turbo Transfer System (High molecular weight, BIO-RAD, Trans-Blot Turbo 1x Transfer Buffer). The immunoblot was blocked in 5% cow milk (diluted in TBS-T) at room temperature for 1h. After 3 washes with 1x TBS-T the HEK293T cell blot was incubated overnight at 4 °C with the following antibodies diluted in 5% Bovine Serum Albumin (Sigma): Myc-Taq (Cell Signaling #2278S, 1:250) and GAPDH (Cell Signaling #5174S, 1:1,000). The leukemia lysate blot was incubated with the following antibodies diluted in 5% bovine serum albumin (Sigma): WAPL (Cell Signaling #77428, 1:1000), RAD21 (Bethyl #A300-080A, 1:10,000), HSP90 (Cell Signaling #4877S, 1:1,000) and β-Actin (Santa Cruz Technology

#B0719, 1:1,000). The Myc-Tag validation blot was incubated with Myc-Tag (Cell Signaling #71D10, 1:250) and β-Actin (Santa Cruz Technology #B0719, 1:1,000). The following day, the secondary antibody depending on primary species was applied after 3 consecutive washes (Cell Signaling Anti-Rabbit IgG #7075 1:1,000, Cell signaling Anti-Mouse IgG #7076) for 1 h in the dark, at room temperature diluted in 5% milk. After 3 consecutive washes the blot was imaged after application of HRP linked solution (SuperSignal West Pico PLUS Chemiluminescent Substrate, Thermofisher). After stripping (Millipore Reblot Plus Strong Solution 10x) and re-blocking with 5% milk, the membrane was incubated with RAD21 (Bethyl #A300-080A 1:10,000) and Lamin B1 (Cell Signaling #12586, 1:1,000).

HEK293T cells stably overexpressing pRTS-1-RAD WT vs RAD21 P.298S and P298A were induced with 2 μg/ml Doxycycline for 72 as previously described (14). After sorting for high EGPF, lysates from $5x10^5$ were prepared with RIPA buffer and further processing was performed as described above. Transfer was performed using the Trans-Blot Turbo Transfer System (Mixed molecular weight, BIO-RAD, Trans-Blot Turbo 1x Transfer Buffer). Antibodies used were against RAD21 (1:10,000; Bethyl) and GAPDH (1:2000; Cell Signaling).

Immunoprecipitation (R32-hRAD21):

HEK293T cells stably overexpressing R32-hRAD21 WT, p.P298S or p.P298A were

arrested in the metaphase by colchichine. Cells growing exponentially were FCS deprived for 24 h (cultured only in DMEM + 1% Penicillin/Streptomycin) and then treated with 5 µg/ml colchicine (dissolved in DMSO). After 2 h, all cells were collected and washed 3x with PBS. Nuclear lysates were prepared using the NE-PER Nuclear and Cytoplasmic Extraction Reagents (Thermofisher). Protein concentration was evaluated using Bradford assay. 100 µg per lysate was used for immunoprecipitation. Immunoprecipitation was performed using RAD21 Antibody (Bethyl concentration: 1,000 µg/ml, 4 µg per 100 µg of lysate) or Myc-Tag (Bethyl #A191-101, concentration 250 µg/ml, 2 µg per 100 µg of lysate) in 500 µl PBS with freshly added 25x Protease Inhibitor Cocktail (PIC) and 10x PhosSTOP (PS, Roche). Immunoprecipitation samples were rotated overnight at 4 °C. 16 h later 7 µl of each Dynabeads Protein A (Thermofisher #10001D) and Protein G (Thermofisher #10003D) were added and rotated at 4 °C for another 4 h. IP was performed using DynaMac-2 (Thermofisher #12321D) with 3 consecutive washes of PBS/PIC/PS. Immunoprecipitation samples were heated like input samples to 95 °C for 10 min and with 350 rpm rotation. Immunoblotting was performed as described above using 15 µg of nuclear lysate as input control. Flow through and washes were immunoblotted equally for technical validation.

After transfer and blocking, the membrane was incubated with the primary antibodies overnight at 4 °C: RAD21 (Bethyl #A300-080A 1:10,000), Myc-Tag (Bethyl #A191-101, 1:1000), Lamin B1 (Cell Signaling #12586, 1:1,000 or 1:500), SMC1 (Bethyl #A300-055A,

1:1,000), SMC3 (Bethyl #A300-060A, 1:5,000), SA1 (Bethyl #A300-157A, 1:1,000), SA2 (Bethyl #A300-158A, 1:1,000), PDS5B (Bethyl #A300-537A, 1:250), WAPL (Cell Signaling #D9J1U, 1:1,000).

Secondary antibodies were added as described above. Stripping of blots was performed with Reblot Plus Strong Solution (Millipore #2504) for 22 min.

Irradiation and Cell Cycle Analysis:

Fibroblasts were seeded in T25 cm^2 bottles at a density of 1-2x10^5 cells and cultured at 37 °C and 5% CO_2. After reaching 70% confluency cells were irradiated with 6 Gy and 10 Gy. Negative control (0 Gy) was kept outside the incubator in the meanwhile. After 48 h apoptosis was analyzed by propidium iodide staining and flow cytometry adapted from Riccardi and Nicoletti (15). In short: cells were trypsinized (GibcoTrypLE Express), washed with PBS (Dulbecco's Phosphate Buffered Saline), centrifuged at 200 xg for 5 min at RT and fixated with 70% ice-cold Ethanol/PBS for at least 20 min (up to 1 h) at -20 °C. Cells were then centrifuged (all centrifugation steps at 400 xg, 4 °C) and washed with cold PBS. After centrifugation cells were treated with 500 µl of DNA extraction buffer (Nicoletti) and 500 µl of cold PBS. After 5 min incubation on ice cells were again centrifuged and resuspended in staining solution (PBS, 100 µg propidium iodide (BioLegend #421301), 1 mg DNA-free RNAse (Thermo Scientific #EN0531) and 2 mM EDTA (Nicoletti). Cells were incubated protected from light for at least 30 min before

FACS analysis.

Mitomycin-C Treatment and Cell Cycle Analysis:

Fibroblasts were seeded in 10 mm plates or T25 cm^2 bottles at a density of 1-3 x 10^5 cells and cultured at 37 °C and 5% CO_2. After reaching 70% confluency, cells were washed with PBS and treated with Mitomycin-C from *Streptomyces caespitosus* (Sigma #M4287) diluted in PBS to a final concentration of 0.1 mg/ml and incubated at room temperature for 5 min. The negative control was kept in PBS only. After 5 min cells were washed 3x with PBS and cultured with fresh media at 37 °C and 5% CO_2 for 24 h, before apoptosis was analyzed by propidium iodide staining and flow cytometry as explained for "Irradiation and Cell Cycle Analysis".

Immunofluorescence Staining:

Hek293T cells were plated onto Poly-L-Lysine pre-coated coverslips in 24-well-plates at a density of 6-8 x 10^4 cells and cultured for 48 h at 37 °C and 5% CO_2. Cells were fixed for 15 min in 3% formaldehyde/PBS, blocked with 0.25% Triton X-100/PBS and blocked in 1% bovine serum albumin/PBS for 30 min. Samples were incubated with primary antibodies for 1 h at RT. yH2AX and 53BP1 foci were detected using a mouse polyclonal anti-phospho-histone H2AX (Ser139) antibody (Millipore #05-636) at a dilution of 1:100 and a rabbit 53BP1 antibody (Novusbio #NB100-304) at a dilution of 1:1,000. Coverslips

were further stained with secondary antibodies for 1 h at room temperature in the dark. The goat anti-mouse Alexa Fluor 488 IgG antibody (Invitrogen #A-11029) and the goat anti-rabbit Alexa Fluor 594 IgG antibody (Invitrogen #A-11037), were used as secondary antibodies, each at a dilution of 1:200. Slides were mounted in ProLong Diamond Antifade medium containing DAPI. Wide field microscopy was performed with a Zeiss Axio Observer microscope (CFCI, TU Dresden) using a Plan Apochromat objective. The DAPI images were used to detect signals inside the nuclei.

Single-cell RNA Sequencing

Healthy human bone marrow scRNA-seq data from eight donors was downloaded from Human Cell Atlas (https://data.humancellatlas.org/explore/projects/cc95ff89-2e68-4a08-a234-480eca21ce79) (16) and aligned to hg19 using Cell Ranger v3.0.0. Scanpy (https://doi.org/10.1186/s13059-017-1382-0) was used to characterize the cell types in the data, correcting for possible batch effects with Mutual Nearest Neighbors (https://doi.org/10.1038/nbt.4091) and filtering for outliers using median absolute deviation. Cell clusters found with Louvain clustering (https://zenodo.org/record/1054103) were mapped to cell types using known marker genes. Cell cycle phases were annotated by scoring cell cycle marker gene sets from https://doi.org/10.1101/gr.192237.115. Two-dimensional visualization was done with UMAP (https://doi.org/10.1038/nbt.4314).

Supplementary References

1. Bolger AM, Lohse M, Usadel B. Trimmomatic: a flexible trimmer for Illumina sequence data. Bioinformatics. 2014;30(15):2114-20.
2. Li H, Durbin R. Fast and accurate short read alignment with Burrows-Wheeler transform. Bioinformatics. 2009;25(14):1754-60.
3. Li H, Handsaker B, Wysoker A, Fennell T, Ruan J, Homer N, et al. The Sequence Alignment/Map format and SAMtools. Bioinformatics. 2009;25(16):2078-9.
4. Pedersen BS, Quinlan AR. Who's Who? Detecting and Resolving Sample Anomalies in Human DNA Sequencing Studies with Peddy. Am J Hum Genet. 2017;100(3):406-13.
5. Koboldt DC, Zhang Q, Larson DE, Shen D, McLellan MD, Lin L, et al. VarScan 2: somatic mutation and copy number alteration discovery in cancer by exome sequencing. Genome Res. 2012;22(3):568-76.
6. McLaren W, Gil L, Hunt SE, Riat HS, Ritchie GR, Thormann A, et al. The Ensembl Variant Effect Predictor. Genome Biol. 2016;17(1):122.
7. Jian X, Boerwinkle E, Liu X. In silico prediction of splice-altering single nucleotide variants in the human genome. Nucleic Acids Res. 2014;42(22):13534-44.
8. Nakken S, Saveliev V, Hofmann O, Moller P, Myklebost O, Hovig E. Cancer Predisposition Sequencing Reporter (CPSR): A flexible variant report engine for high-throughput germline screening in cancer. Int J Cancer. 2021;149(11):1955-60.
9. Kircher M, Witten DM, Jain P, O'Roak BJ, Cooper GM, Shendure J. A general framework for estimating the relative pathogenicity of human genetic variants. Nat Genet. 2014;46(3):310-5.
10. Mizianty MJ, Peng Z, Kurgan L. MFDp2: Accurate predictor of disorder in proteins by fusion of disorder probabilities, content and profiles. Intrinsically Disord Proteins. 2013;1(1):e24428.
11. Shi Z, Gao H, Bai XC, Yu H. Cryo-EM structure of the human cohesin-NIPBL-DNA complex. Science. 2020;368(6498):1454-9.
12. Finn RD, Clements J, Eddy SR. HMMER web server: interactive sequence similarity searching. Nucleic Acids Res. 2011;39(Web Server issue):W29-37.
13. Kamisetty H, Ovchinnikov S, Baker D. Assessing the utility of coevolution-based residue-residue contact predictions in a sequence- and structure-rich era. Proc Natl Acad Sci U S A. 2013;110(39):15674-9.
14. Zuin J, Dixon JR, van der Reijden MI, Ye Z, Kolovos P, Brouwer RW, et al. Cohesin and CTCF differentially affect chromatin architecture and gene expression in human cells. Proc Natl Acad Sci U S A. 2014;111(3):996-1001.
15. Riccardi C, Nicoletti I. Analysis of apoptosis by propidium iodide staining and flow cytometry. Nat Protoc. 2006;1(3):1458-61.
16. Hay SB, Ferchen K, Chetal K, Grimes HL, Salomonis N. The Human Cell Atlas bone marrow single-cell interactive web portal. Exp Hematol. 2018;68:51-61.
